# Artificial Intelligence–Enhanced Multi-Algorithm R Shiny Application for Predictive Modeling and Analytics: Case Study of Alzheimer Disease Diagnostics

**DOI:** 10.2196/70272

**Published:** 2025-12-30

**Authors:** Han Wenzheng, Edmund F Agyemang, Sudesh K Srivastav, Jeffrey G Shaffer, Samuel Kakraba

**Affiliations:** 1 Department of Biostatistics and Data Science, Celia Scott Weatherhead School of Public Health and Tropical Medicine at Tulane University, 1440 Canal St, New Orleans, LA, 70112, United States, 1 5049882475; 2 Tulane Center for Aging, School of Medicine, Tulane University, New Orleans, LA, United States

**Keywords:** artificial intelligence, AI, Shiny Multi-Algorithm R Tool for Predictive Modeling, SMART-Pred, machine learning, Alzheimer disease, predictive modeling, classification algorithms, disease diagnostics and surveillance

## Abstract

**Background:**

Artificial intelligence (AI) has demonstrated superior diagnostic accuracy compared with medical practitioners, highlighting its growing importance in health care. SMART-Pred (Shiny Multi-Algorithm R Tool for Predictive Modeling) is an innovative AI-based application for Alzheimer disease (AD) prediction using handwriting analysis.

**Objective:**

This study aimed to develop and evaluate a noninvasive, cost-effective AI tool for early AD detection, addressing the need for accessible and accurate screening methods.

**Methods:**

The study used principal component analysis for dimensionality reduction of handwriting data, followed by training and evaluation of 10 diverse AI models, including logistic regression, naïve Bayes, random forest, adaptive boosting, support vector machine, and neural network. Model performance was assessed using accuracy, sensitivity, precision, specificity, *F*_1_-score, and area under the curve (AUC) metrics. The DARWIN (Diagnosis Alzheimer With Handwriting) dataset, comprising handwriting samples from 174 participants (89 patients with AD and 85 healthy controls), was used for validation and testing.

**Results:**

The neural network classifier achieved an accuracy of 91% (95% CI 0.79‐0.97) and an AUC of 94% on the test set after identifying the most significant features for AD prediction. These performance results surpass those of current clinical diagnostic tools, which typically achieve around 81% accuracy. SMART-Pred’s performance aligns with recent AI advancements in AD prediction, such as Cambridge scientists’ AI tool achieving 82% accuracy in identifying AD progression within 3 years, using cognitive tests and magnetic resonance imaging scans. The variables “air_time” and “paper_time” consistently emerged as critical predictors for AD across all 10 AI models, highlighting their potential importance in early detection and risk assessment. To augment transparency and interpretability, we incorporated the principles of explainable AI, specifically using Shapley Additive Explanations, a state-of-the-art method to emphasize the features responsible for our model’s efficacy.

**Conclusions:**

SMART-Pred offers noninvasive, cost-effective, and efficient AD prediction, demonstrating the transformative potential of AI in health care. While clinical validation is necessary to confirm the practical applicability of the identified key variables, the findings of this study contribute to the growing body of research on AI-assisted AD diagnosis and may lead to improved patient outcomes through early detection and intervention.

## Introduction

Artificial intelligence (AI) is revolutionizing medical diagnosis, offering unprecedented accuracy and efficiency in health care [1]. AI algorithms can analyze vast amounts of complex medical data, including images, patient records, and genetic information, to detect diseases earlier and more precisely than traditional methods [1-3]. In some cases, AI outperforms human practitioners in diagnostic accuracy, particularly in fields like radiology and pathology [4-8]. For instance, AI systems can identify subtle patterns in electrocardiograms to detect genetic heart conditions and analyze retinal images to diagnose diabetic retinopathy in its early stages [9-13]. By augmenting human expertise, AI not only improves diagnostic speed and accuracy but also has the potential to extend quality health care to remote areas, ultimately leading to better patient outcomes and more personalized treatment plans [1,3,4,14-17].

Alzheimer disease (AD) is a progressive brain disorder that gradually impairs memory and cognitive abilities, ultimately affecting the capacity to perform even the most basic tasks [18-22]. In most cases, especially those involving late-onset AD, symptoms usually appear in individuals during their mid-60s. Alzheimer disease and related dementias (ADRD) represent the primary cause of dementia among older adults [23-26]. In contrast, early-onset ADRD, which is relatively uncommon, can develop in people aged 30-65 years [27]. Aging is common to all cells and organelles in living organisms [28-31]. In recent times, there has been a disproportionate increase in the occurrence of ADRD, Huntington disease, Parkinson disease, amyotrophic lateral sclerosis, and other neurodegenerative diseases [32-35]. It is estimated that about 50 million people across the globe are living with ADRD [36,37]. ADRD are associated with a large family and caregiver burden. In 2018, the United States spent as much as US $277 billion on care for patients with ADRD. It is estimated that over 100 million people will be living with ADRD by the year 2050, for which the associated cost of treatment will be US $1200 billion [38-40]. Even though the prevalences of most diseases, such as breast cancer, prostate cancer, heart disease, stroke, and HIV, have decreased from 2000 to 2015, the prevalence of ADRD has increased by 123% within the same period [19].

Although many interventions, including novel nonsteroidal anti-inflammatory drugs and small molecules, have been explored for the treatment of ADRD [41-44], there is currently no cure for ADRD. Consequently, early diagnosis and the identification of ADRD biomarkers are critical to tailoring appropriate interventions for ADRD, reinforcing the need to design optimal methods for the prediction of ADRD and identify individuals at higher risk of ADRD in a timely manner [14,45-49].

The rapid growth of big data has necessitated the development of robust AI-driven algorithms for predictive modeling [50-58]. These algorithms are essential for efficiently handling large datasets and extracting relevant information through machine learning (ML) techniques [59]. Illustratively, ML algorithms, specifically hierarchical clustering, have been applied to visualize and examine the effects of point mutations that are associated with different phenotypes of diseases, like COVID-19, cystic fibrosis, and sickle cell anemia, resulting in an improved understanding of how point mutations lead to various variants of diseases [60-62]. AI and ML models have also found their place in disease prediction and risk analysis [54,63,64] for various diseases (neurodegenerative disorders, cardiovascular diseases, renal diseases, infectious diseases, diabetes, chronic diseases, neurological and psychiatric disorders, obesity, COVID-19, skin diseases, brain tumors, thyroid diseases, and liver diseases) [64-67]. However, no single algorithm can optimally solve every problem, highlighting the need for diverse ML approaches in predictive modeling and analytics. R, a widely adopted programming language for statistical data analysis, has become a powerful tool in data science [68]. The introduction of the Shiny package by RStudio revolutionized web development within the R ecosystem, making it accessible to both experienced data scientists and those with limited programming experience [69,70].

## Methods

### Development and Framework of the Shiny Multi-Algorithm R Tool for Predictive Modeling Application

SMART-Pred (Shiny Multi-Algorithm R Tool for Predictive Modeling) is an innovative AI-based R Shiny application developed to address the increasing demand for accessible data science tools and to simplify the complexity of ML workflows [67,71,72]. This user-friendly platform automates essential steps, such as data preprocessing, model training, hyperparameter tuning, model validation, and performance evaluation. It features a web interface that allows users to upload and preview datasets, configure variables, and compute descriptive statistics. SMART-Pred incorporates 10 classic ML algorithms, including logistic regression, random forest, adaptive boosting (AdaBoost), support vector machine (SVM), and neural network, for robust classification tasks. The tool also offers interactive data frames for comparing model evaluation metrics, alongside visualization capabilities like multimodel receiver operating characteristic (ROC) curve comparisons, feature importance ranking (by each selected algorithm saved as a CSV file in the same working directory in addition to the graphical display), and the Shapley Additive Explanations (SHAP) framework, which can provide the direction of influence of each feature. With one-click server deployment, SMART-Pred ensures accessibility anytime and anywhere, creating a convenient and intuitive environment for ML modeling that bridges the gap between complex AI algorithms and practical applications, potentially accelerating research and innovation across data-driven fields.

The DARWIN (Diagnosis Alzheimer With Handwriting) dataset [73] was used to validate SMART-Pred’s classification capabilities and benchmark its performance against prior studies. Specifically curated for early AD detection, the DARWIN dataset comprises handwriting data from 174 participants (89 patients with AD and 85 healthy controls) across 452 columns, including unique identifiers, classification labels (“P” for patient and “H” for healthy), and 451 features extracted from 25 structured tasks (graphic, copy, memory, and dictation exercises) with 18 features per task. Collected using standardized protocols (eg, Wacom tablets at 200 Hz), this dataset captures fine motor and cognitive variations linked to neurodegeneration, offering a balanced sample to minimize algorithmic bias and richer multidimensional data compared to single-task datasets. Unlike neuroimaging-focused alternatives like Alzheimer’s Disease Neuroimaging Initiative (ADNI), the DARWIN dataset supports noninvasive, cost-effective, and immediately deployable screening in clinical settings, aligning with SMART-Pred’s goal of accessible early AD detection [73,74]. Its feature richness and task diversity mitigate overfitting risks and provide direct insights into cognitive-motor integration, a hallmark of AD progression, while prioritizing actionable, low-resource biomarkers over complex biological data, thus enabling it to offer pragmatic solutions for early diagnosis in diverse health care environments. The SMART-Pred application and data are provided in Multimedia Appendix 1.

**Figure 1.**
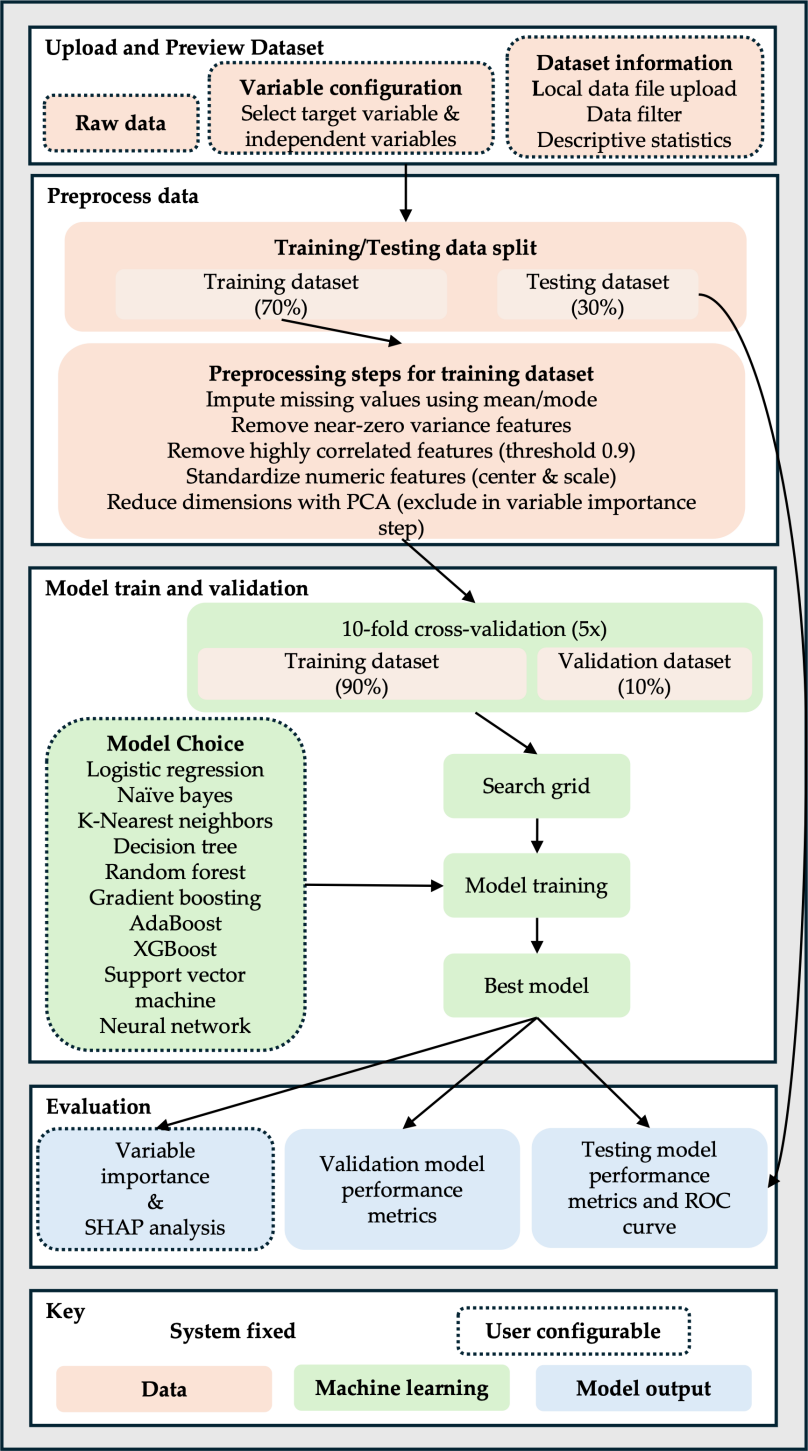
Schematic workflow of the SMART-Pred (Shiny Multi-Algorithm R Tool for Predictive Modeling) application. The comprehensive workflow of the SMART-Pred application is organized into four principal stages: (1) uploading and previewing the dataset, (2) preprocessing data, (3) model training and validation, and (4) model evaluation. Graphical elements indicate process types: dotted boxes represent user-configurable steps (eg, data selection, feature handling, and model settings), while solid boxes denote system-fixed operations. The color scheme clarifies each stage’s nature: pink for data-related processes, green for machine learning operations, and blue for model outputs. This visual summary highlights both the modular structure and the flexible customization available within SMART-Pred for streamlined, transparent predictive analytics. AdaBoost: adaptive boosting; PCA: principal component analysis; ROC: receiver operating characteristic; SHAP: Shapley Additive Explanations; XGBoost: extreme gradient boosting.

The ultimate objective of this study is to leverage handwriting characteristics from the DARWIN dataset to distinguish between patients with AD and healthy individuals, contributing to earlier, more accessible diagnostic methods that can inform tailored interventions. We hypothesized that the integration of user-configurable and automated ML methods within the SMART-Pred workflow will enable the reliable classification of patients with AD versus healthy individuals based on handwriting features from the DARWIN dataset, demonstrating the potential of handwriting-based digital biomarkers for early, scalable AD detection. SMART-Pred’s end-to-end ML workflow for classification tasks is structured across 4 core components: data input and preview, data preprocessing, model training and validation, and model evaluation. This framework integrates user-configurable options with automated processing to ensure flexibility and methodological rigor. The process starts with data upload and preprocessing, advances through model training with multiple algorithmic choices, and culminates in comprehensive performance evaluation and visualization, as detailed in the flowchart presented in Figure 1. By democratizing access to advanced ML techniques through an intuitive interface, SMART-Pred aims to enhance research capabilities and clinical applicability in AD detection and beyond.

### SHAP-Based Interpretability Method

SHAP is a model-agnostic interpretability technique grounded in cooperative game theory, which quantifies how much each feature contributes to a model’s prediction by computing Shapley values, ensuring consistent and locally accurate explanations for individual outcomes. In its general, model-agnostic form, SHAP approximates these values using a weighted linear regression approach (Kernel SHAP), though specialized algorithms exist for specific model types. SHAP is applied to interpret predictions from all 10 ML classifiers trained on AD data, revealing the relative impact of each input feature on classification outcomes [75,76]. The explanation model g approximates the original complex model f by representing the prediction as a linear combination of feature coalitions given by:


$$g(z^{\prime })=\phi_{0}+\sum_{i=1}^{N}\phi_{i}z_{i}^{\prime },\;z_{i}^{\prime }\in \{0,1{\}}^{N}$$

where $z^{`}$ denotes a binary vector indicating the presence or absence of features in the coalition, N is the total number of AD features, and $\phi_{i}$ corresponds to the Shapley value, reflecting the individual feature’s contribution [77].

Through optimization, the Shapley value $\phi_{i}$ provides a theoretically grounded and consistent measure of feature importance, enhancing the interpretability of the ML classifier’s predictions.

### Data Input and Preview

**Figure 2.**
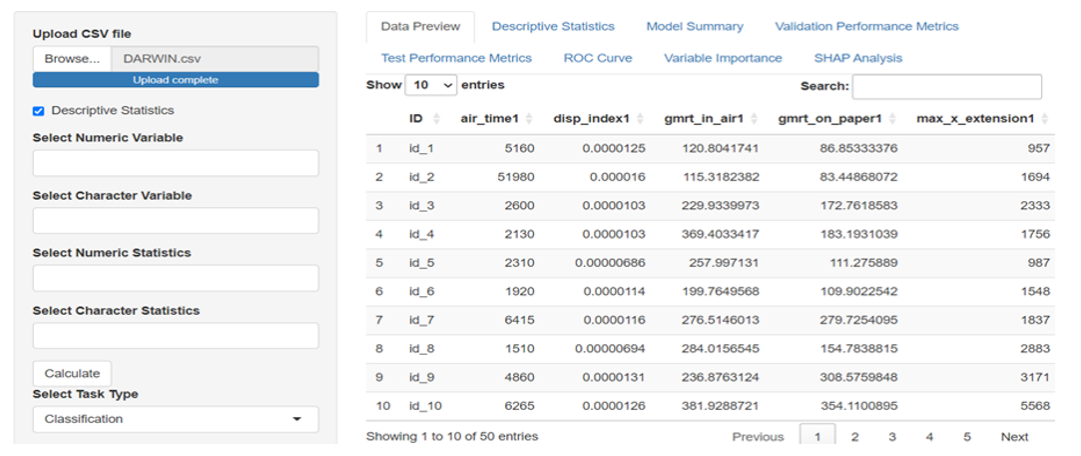
Data input and preview interface of SMART-Pred (Shiny Multi-Algorithm R Tool for Predictive Modeling). The left panel shows data input controls, including CSV file upload. The right panel displays an interactive data preview table with pagination controls (10 entries per page) and search functionality. The interface enables users to configure analytic parameters while maintaining an overview of the dataset structure. ROC: receiver operating characteristic; SHAP: Shapley Additive Explanations.

**Figure 3.**
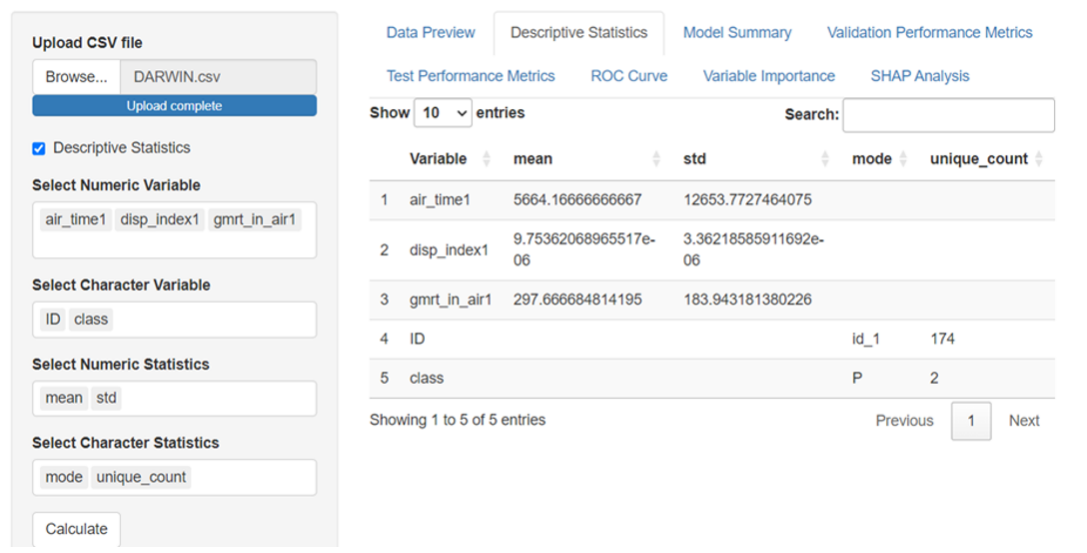
Descriptive statistics interface of SMART-Pred (Shiny Multi-Algorithm R Tool for Predictive Modeling). The left panel includes variable and statistics selection controls: separate boxes for numeric variables (eg, air_time1 and disp_index1) and character variables (eg, ID and class), along with corresponding statistics selection boxes (mean and std for numerical variables; mode and unique count for character variables) and a “Calculate” button to run the process. The right panel displays the calculated statistics in a paginated table format with sortable columns, showing descriptive measures for all selected variables. The interface includes navigation tabs at the top for accessing different analysis views and a search functionality for filtering results. ROC: receiver operating characteristic; SHAP: Shapley Additive Explanations.

**Figure 4.**
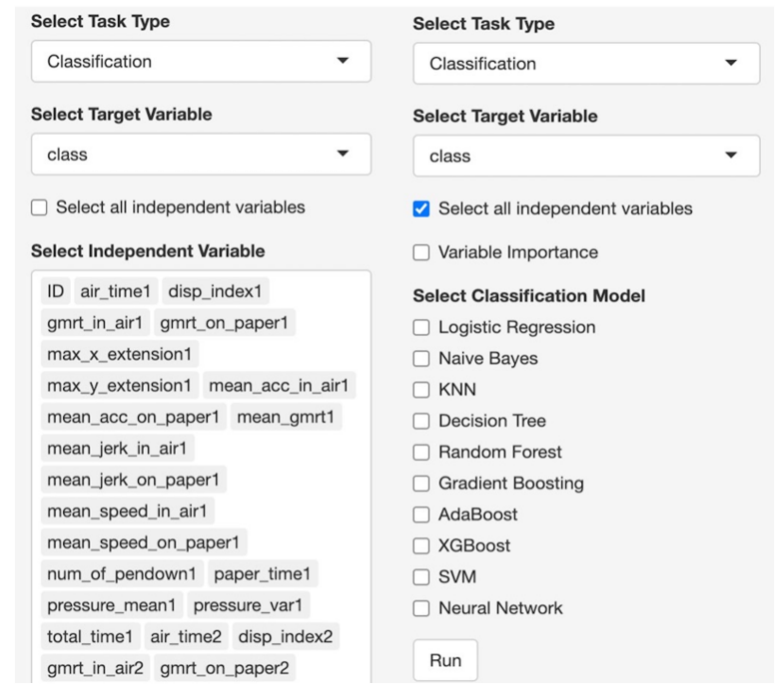
Task and variable selection interface of SMART-Pred (Shiny Multi-Algorithm R Tool for Predictive Modeling). The interface consists of 3 main components: a task type dropdown menu for selecting between classification and regression analysis, a target variable dropdown menu for specifying the dependent variable (eg, “class”), and a scrollable selection box displaying all available independent variables. Users can either select individual variables or use the “Select all independent variables” checkbox for batch selection. The interface displays variable names in a structured format, allowing users to view and select from all the features (eg, air_time1, disp_index1, and gmrt_in_air1). AdaBoost: adaptive boosting; KNN: k-nearest neighbors; SVM: support vector machine; XGBoost: extreme gradient boosting.

**Figure 5.**
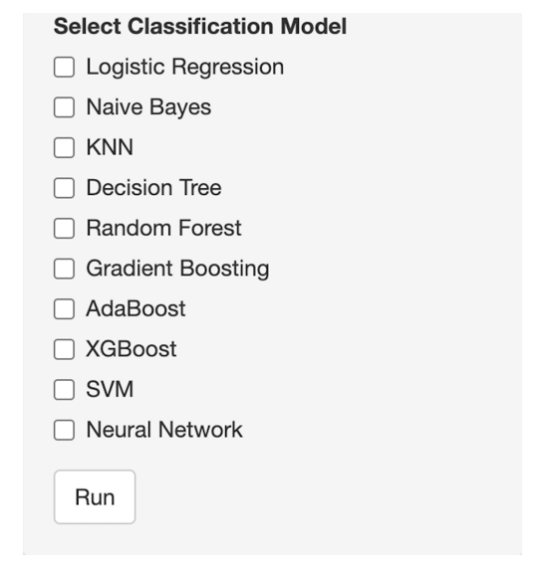
Model selection panel of SMART-Pred (Shiny Multi-Algorithm R Tool for Predictive Modeling). The interface provides checkboxes for selecting from 10 machine learning models: logistic regression, naïve Bayes, k-nearest neighbors (KNN), decision tree, random forest, gradient boosting, adaptive boosting (AdaBoost), extreme gradient boosting (XGBoost), support vector machine (SVM), and neural network. A “variable importance” option enables feature significance analysis, a “SHAP analysis” option estimates Shapley Additive Explanations (SHAP) values to emphasize the direction of the features responsible for the model’s efficacy, and a “Run” button initiates the model training process. The panel allows multiple model selection for comparative analysis.

SMART-Pred allows users to upload local data files through a web interface. Upon uploading, users can preview the dataset in an interactive table format (Figure 2) and access basic descriptive statistics (Figure 3). For descriptive statistics, the interface provides options for variable and measure configuration, where users can choose specific statistical measures: mean, SD, minimum, median, and maximum for numerical variables, and mode and unique count for character variables. After data upload, users proceed to configure the analysis parameters through a structured variable selection process (Figure 4). First, users specify the analysis type by selecting either “Classification” or “Regression” from the task type dropdown menu. For the target variable selection, users choose the dependent variable that represents the outcome to be predicted. The interface then presents a comprehensive list of available independent variables in a scrollable selection box, including various measurement features and other kinematic parameters. Users can either select individual variables or use the “Select all independent variables” checkbox for comprehensive analysis. This structured approach ensures proper specification of the prediction task and relevant features for subsequent analysis. The interface also provides checkboxes for selecting from 10 ML models (Figure 5).

### Data Preprocessing

The preprocessing pipeline (Figure 1) implements a systematic approach to data preparation. The preprocessing pipeline in SMART-Pred begins with a stratified 70‐30 split to preserve class balance between patients with AD and controls, ensuring representative distributions in the training (70%) and test (30%) sets. For missing values, mean/mode imputation is systematically applied: numerical features use subgroup-specific means (eg, imputing based on AD stage or age cohort) to retain biological context, while categorical variables use mode replacement or an “Unknown” category to avoid information loss. This approach balances simplicity with clinical relevance, as arbitrary deletion would disproportionately affect smaller AD subgroups.

Principal component analysis (PCA) was strategically implemented to address the DARWIN dataset’s high dimensionality (451 features), focusing on spatiotemporal handwriting variables like *air_time* and *paper_time*. PCA condensed these into orthogonal components that captured 95% cumulative variance, eliminating redundant kinematic features (eg, overlapping stroke velocity metrics) while preserving discriminative power for AD-related motor-cognitive patterns. The threshold of 95% variance retention was chosen to minimize noise from minor handwriting variations unrelated to neurodegeneration. Prior to PCA, feature filtering removed near-zero variance metrics (<5% unique values) and highly correlated variables (Pearson *r*>0.9), reducing multicollinearity that could distort component interpretation.

For non-DARWIN datasets with missing values, SMART-Pred uses context-aware imputation: numerical features use median values stratified by diagnostic status to avoid dilution of AD-specific patterns, while categorical variables adopt a hybrid approach-mode replacement for features with <10% missingness and “Missing” flagging for higher rates. This dual strategy ensures robustness across heterogeneous data sources while maintaining compatibility with the tool’s multi-algorithm framework. The preprocessing workflow optimizes computational efficiency without sacrificing clinical interpretability, enabling focused analysis of neurodegeneration-sensitive handwriting biomarkers.

### Model Training and Validation

The model training process uses a robust validation strategy involving 10-fold cross-validation repeated 5 times. The system has 10 classic ML algorithms (Table 1). The models range from traditional approaches (eg, logistic regression and naïve Bayes) to advanced ensemble methods (eg, random forest and gradient boosting) and deep learning (eg, neural network), and the selection has been driven by the need to balance algorithmic diversity, clinical utility, and technical robustness. These models have been chosen to cover a broader spectrum of approaches: linear models (eg, logistic regression) provide interpretability for clinical stakeholders, ensemble methods (eg, random forest and AdaBoost) address nonlinear relationships while reducing overfitting, and deep learning models (eg, neural network) capture complex interactions. Criteria, such as accuracy, computational efficiency, and explainability, have guided the selection. Domain-specific validation ensures that the models excel at analyzing temporal handwriting dynamics and mitigates class imbalance. The inclusion of both high-precision neural networks and interpretable models optimizes the tradeoff between complexity and clinical transparency, while cross-validation confirms consistent performance. This multi-algorithm strategy ensures adaptability to diverse patient profiles and aligns with SMART-Pred’s goal of delivering a robust, deployable tool for early AD detection. Detailed information about each algorithm’s implementation and theoretical foundation has been provided previously [25-33]. Hyperparameter optimization is performed through a grid search process to identify the best model configuration.

**Table 1.** Machine learning models implemented in the SMART-Pred^a^ application.

Algorithm	R function	Reference
Logistic regression	glm	[78]
Naïve Bayes	Naïve_bayes	[79]
K-nearest neighbor	knn	[80]
Decision tree	rpart	[81]
Random forest	rf	[82]
Gradient boosting	gbm	[83]
Adaptive boosting	ada	[84]
Extreme gradient boosting	xgbTree	[85]
Support vector machine	svmRadial	[80]
Neural network	nnet	[86]

a SMART-Pred: Shiny Multi-Algorithm R Tool for Predictive Modeling.

### Evaluation Output

**Figure 6.**
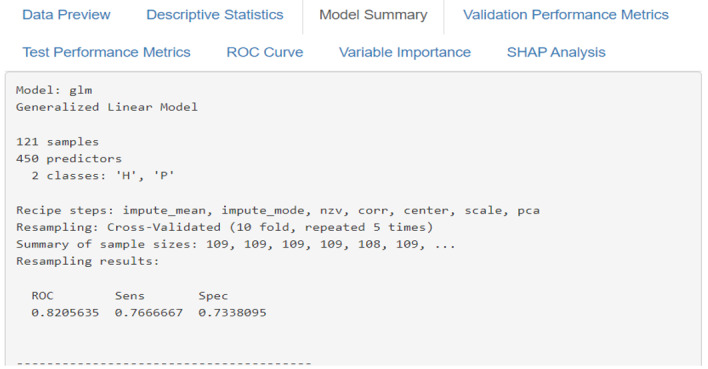
Model summary interface of SMART-Pred (Shiny Multi-Algorithm R Tool for Predictive Modeling) displaying detailed model training results. The interface presents comprehensive information for each trained model and an error message if a problem occurs during model training, including model type (eg, generalized linear model and naïve Bayes), dataset characteristics (eg, 121 samples and 451 predictors), preprocessing steps (eg, impute_mean, impute_mode, nzv, corr, center, scale, and pca), and cross-validation details (10-fold, repeated 5 times). Performance metrics are displayed in a structured format, showing receiver operating characteristic (ROC), sensitivity, and specificity values for each model’s cross-validation results. SHAP: Shapley Additive Explanations.

The evaluation module provides a comprehensive assessment through multiple metrics and visualizations. The system generates validation model performance metrics (Figures6  7), including accuracy [87], sensitivity [88], specificity [88], and *F*_1_-score [89], for model selection; test model performance metrics (Figure 8), including accuracy [34], sensitivity [35], specificity [35], *F*_1_-score [36], and ROC [37], for final evaluation; and ROC curves (Figure 9) for comparing model performance. Additionally, variable importance analysis (Figure 10) is conducted to identify key predictive features across different models. Lastly, SHAP analysis is conducted to interpret the predictions made by the 10 ML classifiers. All evaluation results are presented through interactive visualizations and data frames, allowing the examination of model performance from multiple perspectives.

**Figure 7.**
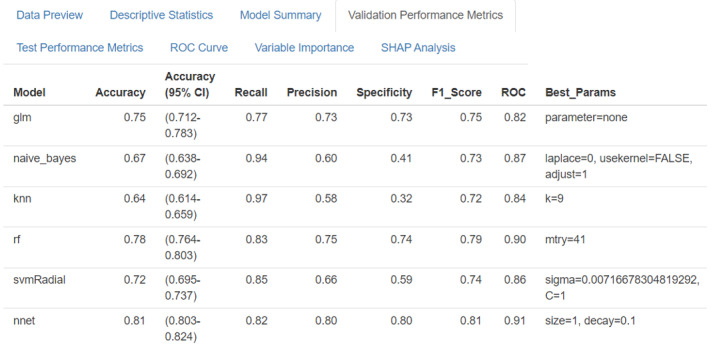
Validation performance metrics comparison interface of SMART-Pred (Shiny Multi-Algorithm R Tool for Predictive Modeling). The table displays comprehensive evaluation metrics for 6 selected machine learning models (logistic regression, naïve Bayes, k-nearest neighbors, random forest, support vector machine, and neural network), including accuracy, recall, precision, specificity, *F*_1_-score, and receiver operating characteristic (ROC) value. Each model’s optimized hyperparameters are shown in the Best_Params column. The interface is accessible through the “Validation Performance Metrics” tab, allowing users to compare model performance across multiple evaluation criteria. SHAP: Shapley Additive Explanations.

**Figure 8.**
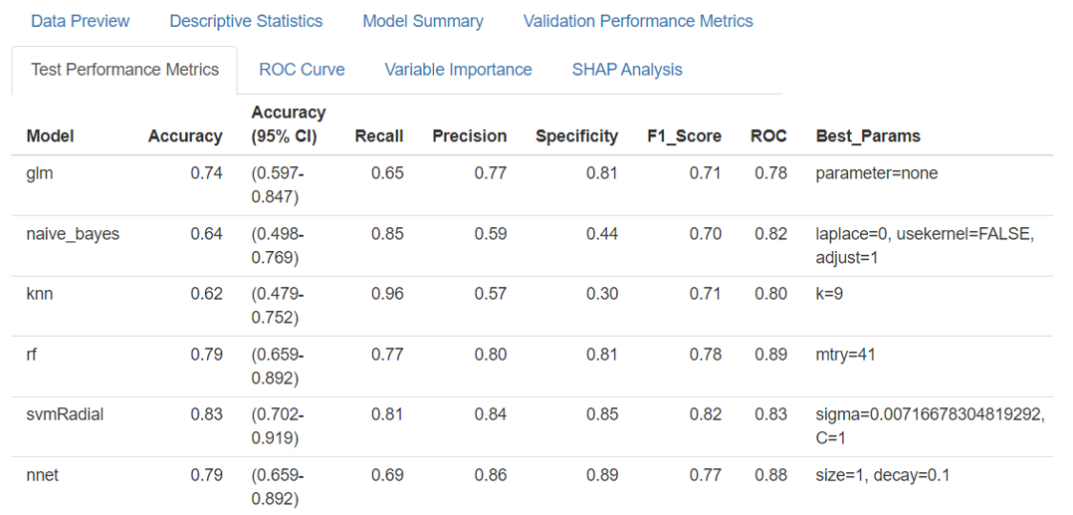
Test performance metrics comparison interface of SMART-Pred (Shiny Multi-Algorithm R Tool for Predictive Modeling). The table displays comprehensive evaluation metrics for 6 machine learning models (logistic regression, naïve Bayes, k-nearest neighbors, random forest, support vector machine, and neural network), including accuracy, recall, precision, specificity, *F*_1_-score, and receiver operating characteristic (ROC) values. Each model’s optimized hyperparameters are shown in the Best_Params column. The interface is accessible through the “Test Performance Metrics” tab, allowing users to compare model performance across multiple evaluation criteria. SHAP: Shapley Additive Explanations.

**Figure 9.**
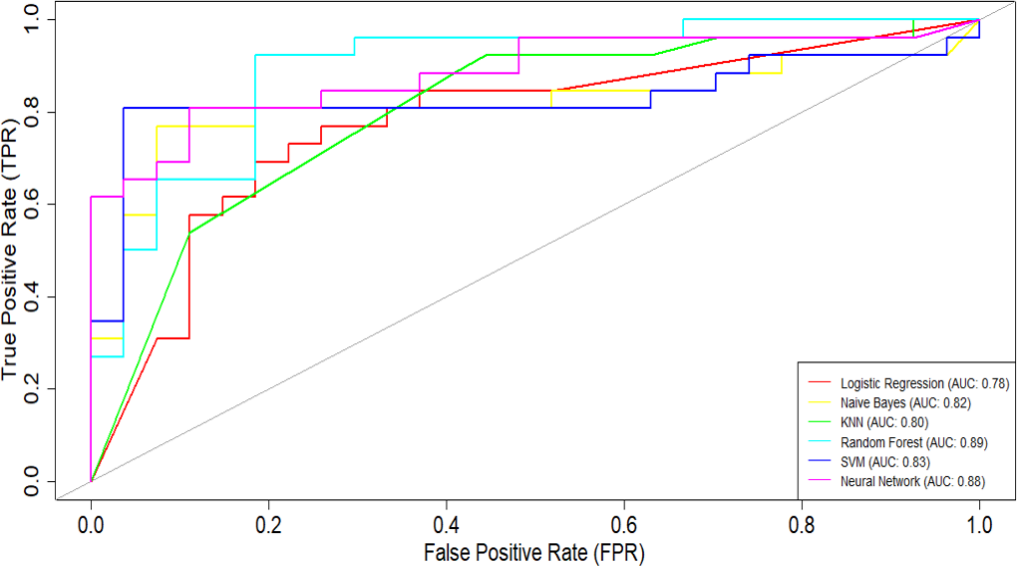
Receiver operating characteristic (ROC) curve comparison interface of SMART-Pred (Shiny Multi-Algorithm R Tool for Predictive Modeling). The plot displays the true positive rate against the false positive rate for 6 classification models, with each model represented by a different colored line. The diagonal reference line represents random prediction (area under the curve [AUC]=0.5). The interface is accessible through the “ROC Curve” tab, allowing users to compare model performance across multiple evaluation criteria. KNN: k-nearest neighbors; SVM: support vector machine.

**Figure 10.**
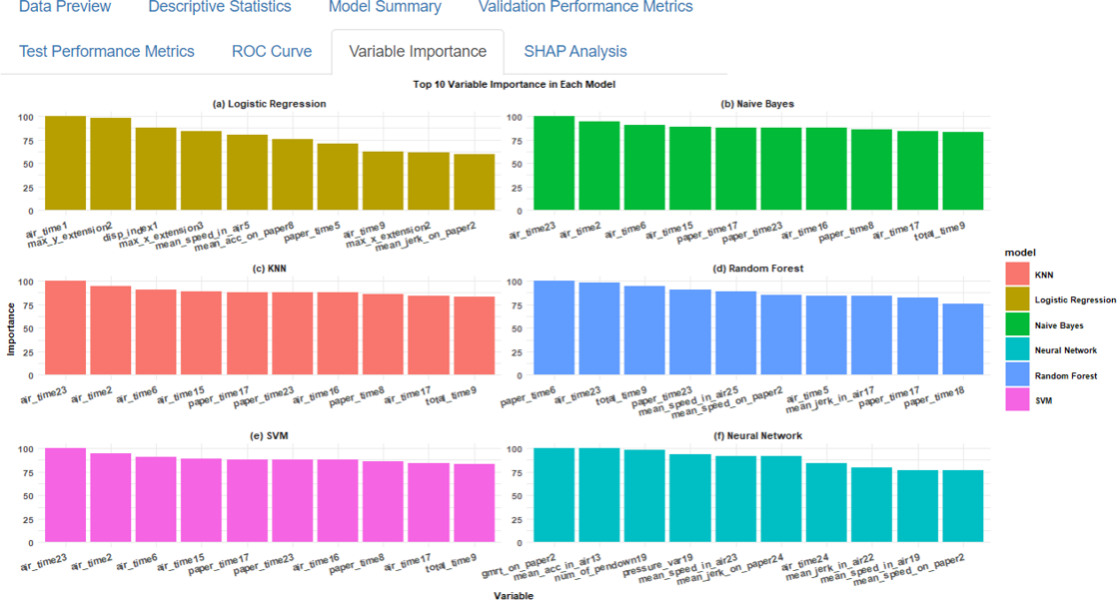
Top 10 variable importance visualization interface in SMART-Pred (Shiny Multi-Algorithm R Tool for Predictive Modeling). The display consists of panels representing different models (eg, logistic regression, naïve Bayes, k-nearest neighbors [KNN], random forest, support vector machine [SVM], and neural network), with each showing bar charts of variable importance scores (y-axis, 0-100 scale) for the most influential predictors (x-axis). Each model type is distinguished by a unique color scheme, allowing for cross-model comparison of feature importance patterns. The interface is accessible through the “Variable Importance” tab, allowing users to compare variable importance across multiple evaluation criteria. ROC: receiver operating characteristic; SHAP: Shapley Additive Explanations.

### Ethical Considerations

This study used secondary, deidentified data and therefore did not require Institutional Review Board approval. All data used in this study have been anonymized to protect privacy.

## Results

### Preliminary Findings

In SMART-Pred, the validation results (Table 2) demonstrated the diverse array of models analyzed, revealing significant variations in performance metrics, such as accuracy, sensitivity, specificity, *F*_1_-score, and area under the curve (AUC), for the DARWIN dataset among the models. The neural network and AdaBoost classifiers emerged as top performers, exhibiting high accuracy scores of 81% and 80%, respectively. Moreover, AdaBoost and neural network achieved commendable AUC scores (92% and 91%, respectively), suggesting a robust capability to distinguish between positive and negative AD cases. Conversely, models, such as k-nearest neighbors (KNN) and naïve Bayes, were noted for lower overall performance, revealing potential limitations in handling the DARWIN dataset’s intricacies.

**Table 2.** Validation performance metric comparison across the 10 machine learning models in the SMART-Pred^a^ application for the full feature model.

Model name	Accuracy, value (95% CI)		Recall	Precision	Specificity	*F*_1_-score	AUC^b^
Logistic regression	0.75 (0.71‐0.78)		0.77	0.73	0.73	0.75	0.82
Naïve Bayes	0.67 (0.64‐0.69)		0.94	0.60	0.41	0.73	0.87
K-nearest neighbors	0.64 (0.61‐0.66)		0.97	0.58	0.32	0.72	0.84
Decision tree	0.69 (0.67‐0.71)		0.58	0.73	0.79	0.65	0.80
Random forest	0.78 (0.76‐0.80)		0.83	0.75	0.74	0.79	0.90
Gradient boosting	0.80 (0.79‐0.82)		0.81	0.80	0.80	0.80	0.91
Adaptive boosting	0.80 (0.79‐0.81)		0.83	0.78	0.77	0.80	0.92
Extreme gradient boosting	0.80 (0.79‐0.80)		0.80	0.79	0.79	0.79	0.91
Support vector machine	0.72 (0.70‐0.74)		0.85	0.66	0.59	0.74	0.86
Neural network	0.81 (0.80‐0.82)		0.82	0.80	0.80	0.81	0.91

a SMART-Pred: Shiny Multi-Algorithm R Tool for Predictive Modeling.

b AUC: area under the curve.

### Full Model With All Features

When the model was used for prediction in the test set (Table 3), the AdaBoost classifier stood out with a remarkable accuracy of 85%, a balanced sensitivity of 88%, an *F*_1_-score of 85%, and an AUC of 92%, illustrating its ability to generalize well beyond the validation dataset. Such consistency between the validation and test phases highlights the robustness of the AdaBoost model for practical applications. The extreme gradient boosting (XGBoost) classifier also demonstrated reliable performance across the dataset, maintaining stable accuracy and specificity, further reinforcing its candidacy for real-world deployment. Many other algorithms in our pipeline, such as the gradient boosting and random forest classifiers, achieved commendable evaluation scores across the evaluation metrics, reaffirming the robustness of the pipeline for classification-based predictive modeling.

**Table 3.** Test performance metric comparison across the 10 machine learning models in the SMART-Pred^a^ application for the full feature model.

Model name	Accuracy, value (95% CI)		Recall	Precision	Specificity	*F*_1_-score	AUC^b^
Logistic regression	0.74 (0.60‐0.85)		0.65	0.77	0.81	0.71	0.78
Naïve bayes	0.64 (0.50‐0.77)		0.85	0.59	0.44	0.70	0.82
K-nearest neighbors	0.62 (0.48‐0.75)		0.96	0.57	0.30	0.71	0.80
Decision tree	0.72 (0.58‐0.83)		0.77	0.69	0.67	0.73	0.73
Random forest	0.79 (0.66‐0.89)		0.77	0.80	0.81	0.78	0.89
Gradient boosting	0.85 (0.72‐0.93)		0.81	0.88	0.89	0.84	0.89
Adaptive boosting	0.85 (0.72‐0.93)		0.88	0.82	0.81	0.85	0.92
Extreme gradient boosting	0.81 (0.68‐0.91)		0.73	0.86	0.89	0.79	0.88
Support vector machine	0.83 (0.70‐0.92)		0.81	0.84	0.85	0.82	0.83
Neural network	0.79 (0.66‐0.89)		0.69	0.86	0.89	0.77	0.88

a SMART-Pred: Shiny Multi-Algorithm R Tool for Predictive Modeling.

b AUC: area under the curve.

On integrating findings from both evaluation phases, it becomes evident that ensemble models (random forest, gradient boosting, AdaBoost, and XGBoost) hold significant promise due to their balanced performance metrics and strong generalization capabilities [90-94]. These models provide a compelling combination of sensitivity and specificity, which is crucial for the diagnostics required in AD detection. The observed fluctuations in the performance of the neural network classifier from validation to testing (especially for recall) suggest that while it possesses inherent strengths, there is room for enhancement.

Our comparative analysis underscores ensemble models (random forest, gradient boosting, AdaBoost, and XGBoost) as the most robust candidates for AD diagnostics in the scope of this work, given their superior performance across both the validation and test datasets [95-98]. These ensemble models have good, balanced accuracy in this regard, making them particularly suited for clinical settings where precise prediction and identification are critical. The insights garnered from this study pave the way for continued exploration into model optimization, potentially enhancing the applicability and accuracy of ML techniques in terms of advancing AD research and the early diagnosis of other diseases, which can be considered crucial in tailoring interventions to target diseases.

**Figure 11.**
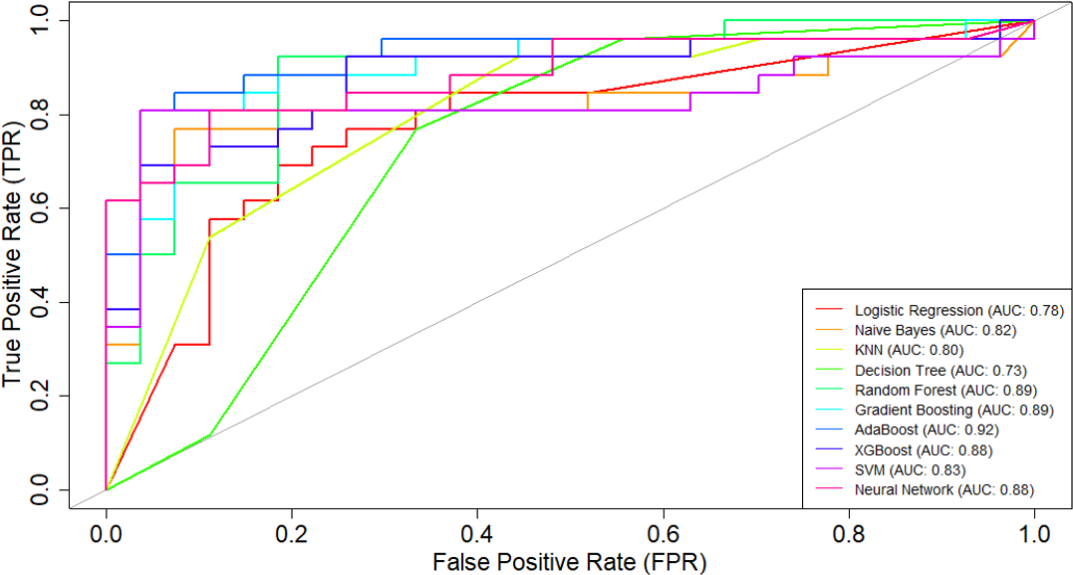
Receiver operating characteristic (ROC) curves across different machine learning (ML) models obtained from SMART-Pred (Shiny Multi-Algorithm R Tool for Predictive Modeling). The plot illustrates the relationship between the true positive rate (y-axis) and false positive rate (x-axis) across 10 different ML models. Each colored curve represents a distinct model’s performance, with the area under the curve (AUC) values shown in the legend. The diagonal gray line represents the random classifier baseline (AUC=0.5). The closer the ROC curve is to the upper left corner of the graph, the higher is the accuracy of the test, as in the upper left corner, sensitivity is 1 and the false positive rate is 0 (specificity=1). The ideal ROC curve thus has an AUC of 1.0. AdaBoost: adaptive boosting; KNN: k-nearest neighbors; SVM: support vector machine; XGBoost: extreme gradient boosting.

Based on the ROC curves (Figure 11), the ensemble models (AdaBoost: AUC=0.92; random forest: AUC=0.89; gradient boosting: AUC=0.89; XGBoost: AUC=0.88) had the strongest performance among the 10 ML models considered in this study. The findings suggest that the AdaBoost ensemble model is highly effective in correctly identifying positive and negative instances for the given classification task. AdaBoost was closely followed by random forest and gradient boosting, both of which demonstrated impressive AUC values of 0.89. The XGBoost and neural network classifiers both achieved an impressive AUC value of 0.88.

In contrast, the decision tree model exhibited the weakest performance, with an AUC of 0.73. While this is still better than random guessing, it suggests that the decision tree algorithm may not be the most suitable choice. The remaining models, including logistic regression, naïve Bayes, SVM, and KNN, had AUC values ranging from 0.78 to 0.83, indicating reasonably good predictive capabilities. The intersecting nature of the ROC curves highlights the better tradeoffs between different models [99-102]. Certain algorithms may excel at specific levels of the false positive rate, which can be an important consideration when selecting the most appropriate model for the task at hand [63].

**Figure 12.**
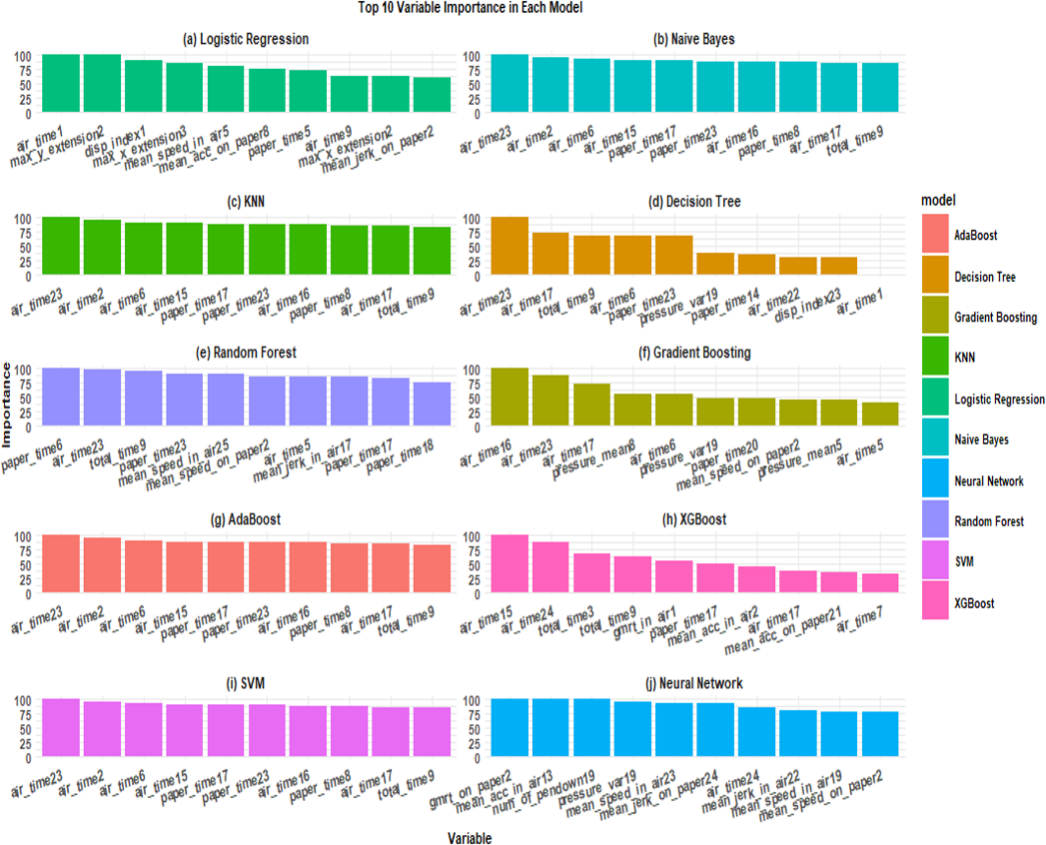
The top 10 variable importance findings across different models obtained from SMART-Pred (Shiny Multi-Algorithm R Tool for Predictive Modeling). The subplots represent the following models: logistic regression, a basic classification algorithm; naïve Bayes, a probabilistic classifier based on Bayes' theorem; k-nearest neighbors (KNN), an instance-based learning algorithm; decision tree, a tree-structured classification model; random forest, an ensemble learning method based on multiple decision trees; gradient boosting, a boosting-based ensemble method; adaptive boosting (AdaBoost), an adaptive boosting algorithm; extreme gradient boosting (XGBoost), an efficient implementation of gradient boosting; support vector machine (SVM), a kernel-based classification algorithm; and neural network, a deep learning architecture. Each subplot represents a specific model's top 10 most important features, with normalized importance scores (y-axis, 0-100) plotted against feature names (x-axis). Different colors distinguish between models, and features are arranged in descending order of importance within each subplot. The importance scores are normalized within each model, with the most important feature scaled to 100.

Building upon our variable importance analysis of SMART-Pred (Figure 12), the partial variable set was constructed by selecting the most influential features among all the algorithms identified through our importance ranking analysis, including “air_time” and “paper_time” variables. Results for the reduced feature model analysis via SMART-Pred are shown in Tables4  5.

**Table 4.** Validation performance metric comparison across the 10 machine learning models in the SMART-Pred^a^ application for the reduced feature model.

Model name	Accuracy, value (95% CI)		Recall	Precision	Specificity	*F*_1_-score	AUC^b^
Logistic regression	0.85 (0.82‐0.88)		0.85	0.85	0.86	0.85	0.89
Naïve Bayes	0.85 (0.83‐0.87)		0.87	0.83	0.84	0.85	0.93
K-nearest neighbors	0.83 (0.81‐0.84)		0.95	0.76	0.71	0.84	0.93
Decision tree	0.82 (0.80‐0.84)		0.77	0.85	0.87	0.81	0.89
Random forest	0.88 (0.87‐0.90)		0.88	0.89	0.89	0.88	0.95
Gradient boosting	0.88 (0.87‐0.89)		0.88	0.88	0.88	0.88	0.94
Adaptive boosting	0.89 (0.88‐0.90)		0.89	0.88	0.88	0.88	0.96
Extreme gradient boosting	0.87 (0.87‐0.88)		0.87	0.87	0.88	0.87	0.94
Support vector machine	0.86 (0.85‐0.88)		0.85	0.87	0.87	0.86	0.93
Neural network	0.86 (0.85‐0.87)		0.87	0.85	0.85	0.86	0.95

a SMART-Pred: Shiny Multi-Algorithm R Tool for Predictive Modeling.

b AUC: area under the curve.

**Table 5.** Test performance metric comparison across the 10 machine learning models in the SMART-Pred^a^ application for the reduced feature model.

Model name	Accuracy, value (95% CI)		Recall	Precision	Specificity	*F*_1_-score	AUC^b^
Logistic regression	0.87 (0.74‐0.95)		0.88	0.85	0.85	0.87	0.88
Naïve Bayes	0.81 (0.68‐0.91)		0.85	0.79	0.78	0.81	0.84
K-nearest neighbors	0.81 (0.68‐0.91)		0.92	0.75	0.70	0.83	0.90
Decision tree	0.81 (0.68‐0.91)		0.88	0.77	0.74	0.82	0.81
Random forest	0.87 (0.75‐0.95)		0.85	0.88	0.89	0.86	0.91
Gradient boosting	0.87 (0.75‐0.95)		0.85	0.88	0.89	0.86	0.90
Adaptive boosting	0.85 (0.72‐0.93)		0.81	0.88	0.89	0.84	0.91
Extreme gradient boosting	0.85 (0.72‐0.93)		0.81	0.88	0.89	0.84	0.87
Support vector machine	0.89 (0.77‐0.96)		0.85	0.92	0.93	0.88	0.92
Neural network	0.91 (0.79‐0.97)		0.88	0.92	0.93	0.90	0.94

a SMART-Pred: Shiny Multi-Algorithm R Tool for Predictive Modeling.

b AUC: area under the curve.

### Reduced Model With Important Features

**Figure 13.**
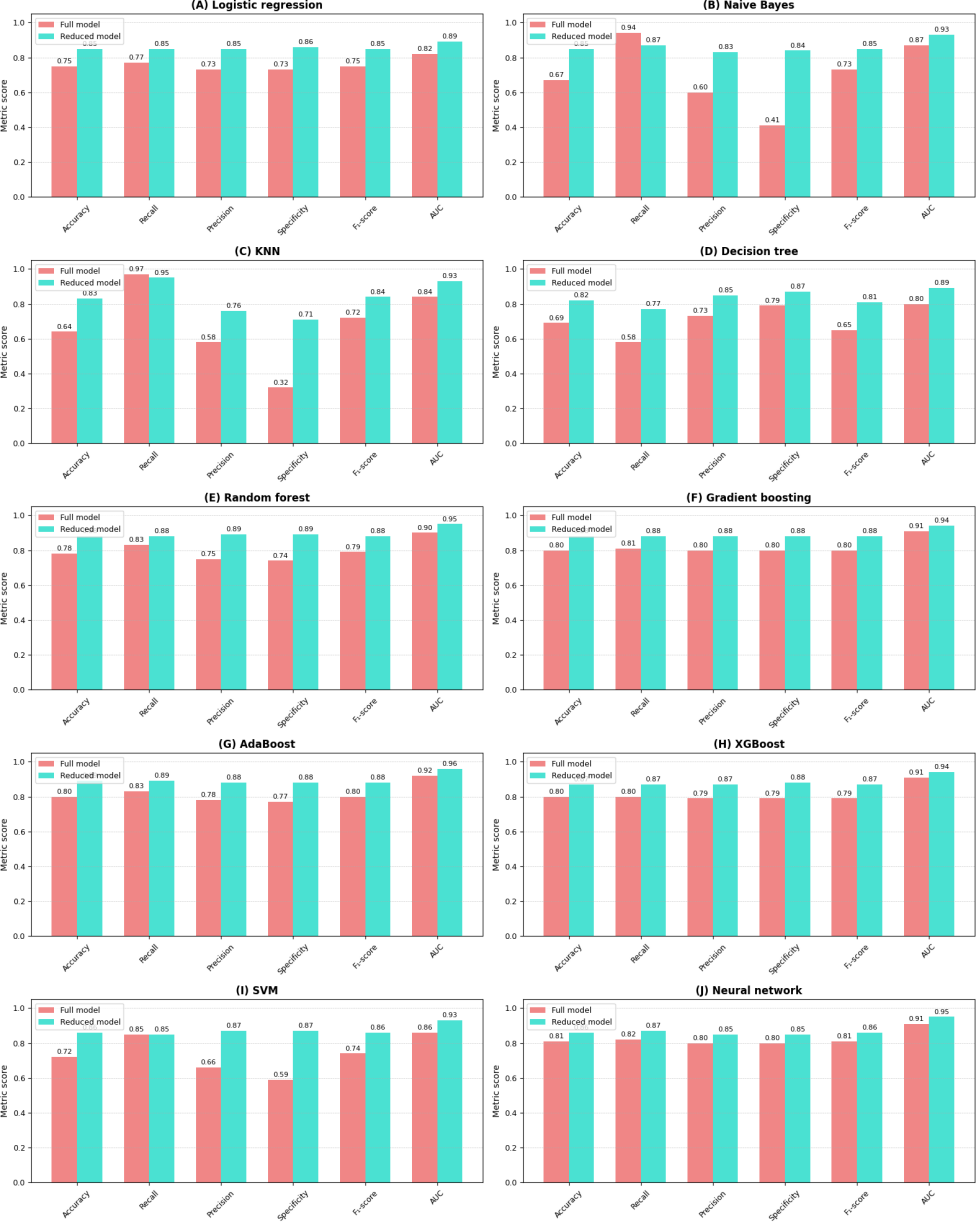
Comparison of validation metrics between the full feature model (all variables included) and the reduced feature model (only the top 2 most important variables across all models in the full feature model) across machine learning models. Each subplot represents a different model, with 6 standardized performance metrics (accuracy, recall, precision, specificity, *F*_1_-score, and area under the curve [AUC]) on the x-axis and their corresponding values (0-1.0) on the y-axis. Red bars represent models trained with all variables, while blue bars indicate models trained with the top 2 most important variables (“air_time” and “paper_time”). This visualization enables a direct comparison of model performance between the full and reduced feature sets across multiple evaluation metrics. AdaBoost: adaptive boosting; KNN: k-nearest neighbors; SVM: support vector machine; XGBoost: extreme gradient boosting.

**Figure 14.**
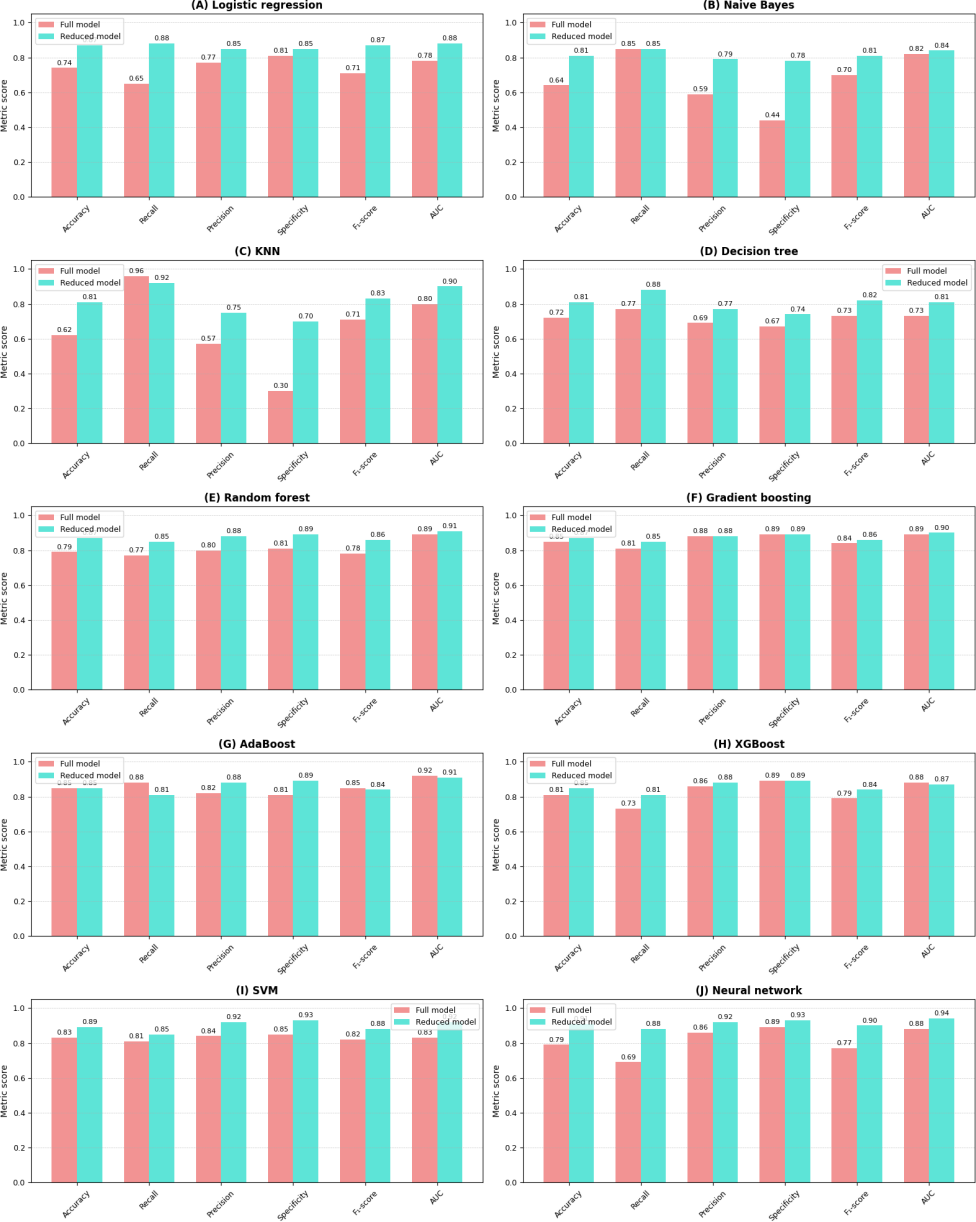
Comparison of test metrics between the full feature model (all variables included) and the reduced feature model (only the top 2 most important variables across all models in the full feature model) across machine learning models. Each subplot represents a different model, with 6 standardized performance metrics (accuracy, recall, precision, specificity, *F*_1_-score, and area under the curve [AUC]) on the x-axis and their corresponding values (0-1.0) on the y-axis. Red bars represent models trained with all variables, while blue bars indicate models trained with the top 2 most important variables (“air_time” and “paper_time”). This visualization enables a direct comparison of model performance between the full and reduced feature sets across multiple evaluation metrics. AdaBoost: adaptive boosting; KNN: k-nearest neighbors; SVM: support vector machine; XGBoost: extreme gradient boosting.

The comparative analysis of the reduced feature model and full feature model showed that models trained on the selected partial variables (reduced feature model) consistently demonstrated equal or superior performance compared to those trained on all variables (full feature model), as seen in Tables 4 and 5. The comparison between the full and partial variable sets (reduced feature model) revealed patterns in both validation (Figure 13) and test (Figure 14) metrics.

The pattern was particularly profound for models, such as SVM, naïve Bayes, KNN, and neural network, which showed substantial improvements across all metrics when using the selected important variables (most notable was the neural network’s approximately 20% increment in the reduced feature model’s test recall, from 69% to 88%). Ensemble methods like random forest, gradient boosting, AdaBoost, and XGBoost demonstrated slight improvements in performance across both variable sets, highlighting their inherent robustness regardless of feature selection [103,104]. Notably, the improvements observed in the validation metrics were largely mirrored in the test metrics, indicating that the benefits of using carefully selected important variables generalize well to unseen data.

**Figure 15.**
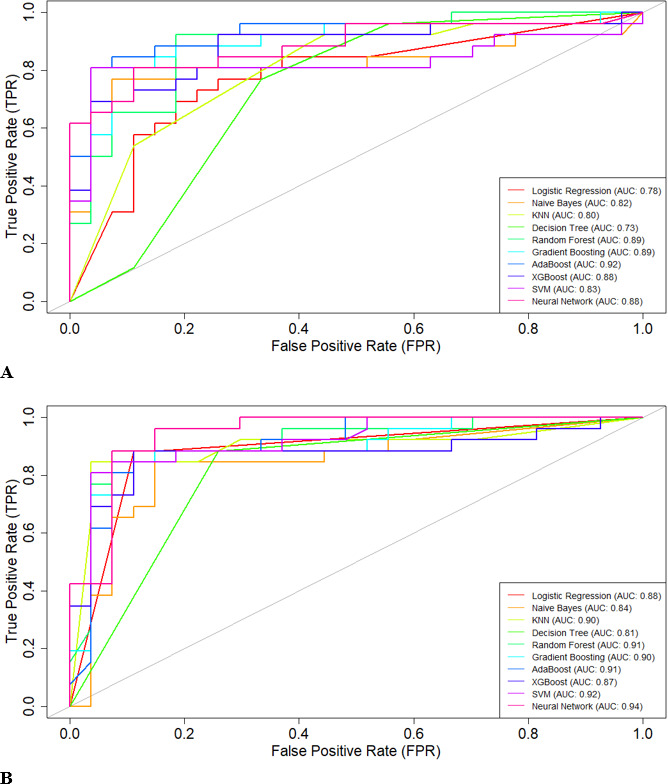
Comparison of receiver operating characteristic (ROC) curves obtained directly from SMART-Pred (Shiny Multi-Algorithm R Tool for Predictive Modeling) between models trained with the full feature set (A) and selected important features (B). Each subplot shows the true positive rate against the false positive rate for 10 machine learning models, with corresponding area under the curve (AUC) values in the legend. Different colors represent different models, and the diagonal gray line indicates random prediction (AUC=0.5). This side-by-side comparison demonstrates the impact of feature selection on model discrimination ability. The closer the ROC curve is to the upper left corner of the graph, the higher is the accuracy of the test, as in the upper left corner, sensitivity is 1 and the false positive rate is 0 (specificity=1). The ideal ROC curve thus has an AUC of 1.0. AdaBoost: adaptive boosting; KNN: k-nearest neighbors; SVM: support vector machine; XGBoost: extreme gradient boosting.

The consistent enhancement across various performance indicators, including accuracy, recall, precision, *F*_1_-score, and ROC values, in both the validation and test scenarios reinforces the effectiveness of our variable importance–based feature selection approach. These findings strongly validate our strategy of using variable importance ranking for feature selection, demonstrating that focusing on the most influential variables not only maintains or improves model performance but also potentially reduces computational complexity and simplifies the model optimization process [105-108]. In the reduced feature model, the SVM classifier achieved an accuracy of 89% (95% CI 0.77‐0.96) with an AUC of 92%, while the neural network algorithm reached an accuracy of 91% (95% CI 0.79‐0.97) with an AUC of 94%, after identifying the most significant features for AD prediction in the test set. The comparison of ROC curves is presented in Figure 15.

### SHAP Analysis on the DARWIN Dataset

Interpreting the results and understanding the decision-making process of ML models has become paramount, especially in critical fields like medical diagnostics. In the context of AD detection, understanding which features or biological markers most influence a model’s prediction (specifically direction of influence) can provide invaluable insights for medical professionals. In lieu of this, this study performed SHAP analysis. However, for brevity and ease of presentation, the SHAP analysis of the 2 best models (neural network and SVM classifiers) is presented in this section, while the SHAP analysis of the remaining 8 models has been presented in Multimedia Appendix 2.

**Figure 16.**
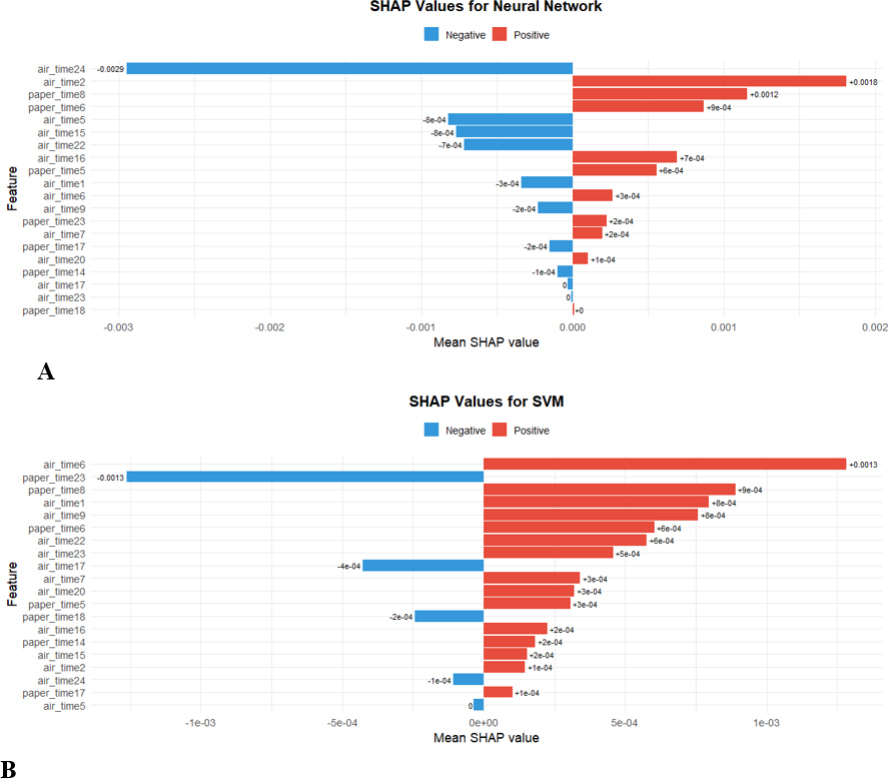
Shapley Additive Explanations (SHAP) summary plots for (A) neural network and (B) support vector machine (SVM) classifiers. Each plot visualizes the impact of individual features on model output using SHAP values, which quantify both the magnitude and direction of each feature’s contribution to the prediction. In these summary plots, features are ranked from top to bottom by their overall importance in the model’s decision-making process. Each point represents a feature value for an instance in the dataset, with color gradients indicating whether the feature value is low (blue) or high (red). The horizontal axis shows the SHAP value, reflecting whether an increased feature value drives the model output higher (positive SHAP value) or lower (negative SHAP value). Comparisons between neural network and support vector classifier results in the figure highlight both consensus and differences in feature importance and decision logic across models. These SHAP visualizations provide insights into which features most influence model predictions, supporting the interpretability and transparency of the machine learning models used in this research.

The SHAP analysis of the neural network classifier indicated that *air_time24* and *air_time5* (mean SHAP values of −0.0029 and −0.0008, respectively) had the greatest negative influence on AD prediction, while *air_time2* and *paper****_****time8* (mean SHAP values of +0.0018 and +0.0012, respectively) had the greatest positive influence on AD prediction (Figure 16A).

For the support vector classifier, *paper_time23* and *air_time17* (mean SHAP values of −0.0013 and −0.0004, respectively) had the greatest negative influence on AD prediction, while *air_time6* and *paper_time8* (mean SHAP values of +0.0013 and +0.0009, respectively) had the greatest positive influence on AD prediction (Figure 16B).

## Discussion

### Robustness of the SMART-Pred Application

AI is transforming medical practice and research by enhancing disease diagnostics, accelerating biomarker discovery, and revolutionizing disease surveillance [109-115]. In diagnostics, AI algorithms can examine complex medical data, such as imaging findings, genetic information, and clinical records, to identify subtle patterns that might be missed by human observers [116-120]. This capability leads to earlier and more accurate disease detection, potentially improving patient outcomes. In biomarker identification, AI’s ability to process vast amounts of multiomic data is uncovering novel indicators of disease presence, progression, and treatment response [121,122]. These AI-discovered biomarkers are paving the way for personalized medicine approaches and more targeted therapeutic interventions. For disease surveillance, AI systems are proving to be invaluable in monitoring and predicting disease outbreaks on a global scale [123]. By analyzing diverse data sources, including social media, environmental factors, and health records, AI can identify emerging health threats in real-time, enabling rapid response and containment strategies [124,125]. Moreover, AI’s impact extends to drug discovery, treatment optimization, and health care resource allocation, promising a future of more efficient, effective, and accessible health care delivery [126,127].

The SMART-Pred application demonstrates a robust, user-friendly, and customizable approach to variable importance analysis across multiple ML models. This innovative tool offers valuable insights into the predictive power of various features in disease diagnostics, biomarker identification, and disease surveillance, particularly in the context of AD, as demonstrated in this study. By using the “varImp()” function and SHP analysis in R in SMART-Pred, our study identified the top-most influential variables for each model, providing a comprehensive view of feature relevance in AD prediction.

### Discussion of the Findings

A thorough analysis of variable importance across all models revealed consistent patterns and model-specific characteristics. The “air_time” features, particularly air_time23, air_time2, and air_time6, emerged as consistently important variables across multiple models, including naïve Bayes, KNN, AdaBoost, and SVM. Additionally, “paper_time” features (paper_time17 and paper_time23) showed significant importance across several models, indicating the crucial role of temporal features in prediction. The selection of air_time (pen movement duration above paper) and paper_time (pen contact duration) in SMART-Pred’s reduced feature model is rooted in their direct biological ties to AD pathology. Air_time reflects dysfunction of the prefrontal cortex, a region critical for motor planning and task initiation [128,129]. AD-related neurodegeneration is reflected in handwriting performance, with patients showing longer overall writing times and substantially increased air times between pen strokes across everyday copying tasks, indicating hesitation and disrupted sequencing of movements as they struggle to translate cognitive intentions into fluent motor output [130-132]. Paper_time, conversely, correlates with motor execution deficits caused by degeneration of the basal ganglia and cerebellar region, which impairs fine motor control. Quantitative tablet-based studies have reported that kinematic and pressure features, such as time in air, speed, and complexity, can correctly classify healthy controls, patients with mild cognitive impairment, and patients with AD, with roughly 70%-80% accuracy in 3-group models and above 90% accuracy in several pairwise contrasts. However, it was observed that the accuracy of classification was significantly influenced by the characteristics of the groups being categorized and the particular task, with rates varying from 63.5% to 100%. Moreover, the diagnostic accuracy derived from kinematic measurements exhibited higher specificity in differentiating normal cognition from impaired cognition (mild cognitive impairment and AD), whereas enhanced sensitivity was seen in discriminating between the degrees of impaired cognition (mild cognitive impairment vs AD) [133-135]. More recent digital pen work has shown that handwriting characteristics distinguish patients with AD from controls, with the task accuracy of the XGBoost model achieving a maximum value of 96.55% and the AUC reaching up to 99.10%, proving that handwriting characteristic analysis is promising in auxiliary AD screening or AD diagnosis [136]. On the other hand, the spatiotemporal dynamics of simple loop handwriting separate patients with early-stage AD from healthy older adults, with about 74% classification accuracy [137]. These findings support total duration, time in air, and related temporal variability as sensitive quantitative markers that differentiate healthy aging, mild cognitive impairment, and AD and that can contribute to early staging and screening.

The validity of the variables is further supported by multimodal evidence. Neuroimaging and digital clock drawing studies have linked temporal handwriting features, including “time in air” and “time on paper,” to global brain and gray matter volumes, while time-based handwriting metrics have shown significant negative correlations with global cognitive scores, such as the MoCA (Montreal Cognitive Assessment), supporting the interpretation of prolonged air_time and paper_time variability as markers of cognitive decline [138-142]. Compared with invasive biomarkers, such as cerebrospinal fluid Aβ42, which require lumbar puncture and typically achieve diagnostic sensitivities of around 80% at a cost of approximately US $1000 per test [143,144], and FDG-PET (fluorodeoxyglucose positron emission tomography) scans, which show similar median sensitivity but are substantially more expensive (often around US $3000 per scan) [145,146], digital clock drawing metrics, such as air_time and paper_time, provide noninvasive, low-cost screening with high diagnostic accuracy for early cognitive impairment and AD [147], with our SMART-Pred protocol achieving 88% sensitivity at a cost of approximately US $5 per test (our study). Their ability to capture both executive dysfunction (via planning delays) and visuospatial deficits (via erratic stroke execution) provides a holistic view of AD’s cognitive-motor integration breakdown, aligning with National Institutes of Health priorities for functional digital biomarkers [148-150]. This dual focus on mechanistic relevance and clinical practicality makes them ideal for scalable, early AD detection in routine care settings.

Consistent with the literature, our findings showed that random forest had a more balanced feature utilization [151,152], with paper_time6 showing maximum importance (100.00), followed by air_time23 (98.27) and total_time9 (94.82). The model also highlighted the significance of diverse features, such as paper_time23 (90.20), mean_speed_in_air25 (89.03), and mean_jerk_in_air17 (84.03), suggesting a more comprehensive feature integration approach [153-156]. Gradient boosting and XGBoost showed similar patterns but with distinct emphases [156]. Gradient boosting prioritized air_time16 (100.00), while XGBoost highlighted air_time15 (100.00) as the most important feature. The gradient boosting model incorporated additional variables like pressure_mean8 and mean_speed_on_paper2 in the predictions, while the XGBoost model incorporated additional variables like gmrt_in_air1 and total_time3 in the predictions. The neural network model exhibited a unique feature importance pattern, emphasizing variables that were less prominent in other models. While including air_time features, it notably prioritized gmrt_on_paper2 (100.00), mean_acc_in_air13 (98.61), and num_of_pendown19 (98.58), suggesting its ability to capture better patterns in the data, particularly those related to acceleration and movement characteristics. These findings regarding variable importance support the notion that interpretability is crucial in AI systems, especially in medical applications [157-161]. The ability to identify and understand underlying diagnostic biomarkers is not only beneficial for enhancing the diagnostic process but also plays a vital role in tailoring treatment plans for patients [162-168].

ROC curve analysis provided a comparative evaluation of various ML models when assessed with all available variables (full feature model) versus a carefully selected subset of the most important variables (“air_time” and “paper_time” variables; reduced feature model). In the full feature model, AdaBoost demonstrated exceptional predictive capability with an AUC of 0.92, while decision tree showed relatively lower performance with an AUC of 0.73. Strong performance was also noted for random forest and gradient boosting (both with an AUC of 0.89) and XGBoost and neural network (both with an AUC of 0.89). In the reduced feature model, neural network and SVM classifiers outperformed all other algorithms (AUC values of 0.94 and 0.92, respectively), revealing their effectiveness even with fewer inputs. Ensemble models, including random forest, gradient boosting, XGBoost, and AdaBoost, continued to perform well, achieving AUC values ranging from 0.87 to 0.91, which reflect their robustness [169-172]. Interestingly, the decision tree’s AUC improved to 0.81, indicating that it benefited significantly from targeted variable selection in the reduced feature model. Consistent with the literature, our results from the SMART-Pred analysis highlight the consistent strength of ensemble models, such as random forest, gradient boosting, XGBoost, and AdaBoost, across varying variable sets and support the strategic approach of leveraging variable importance ranking to enhance model performance [173-179]. By focusing on critical features, models can maintain or even improve accuracy, as demonstrated in this work. However, it should be emphasized that nothing in this work demonstrated causality between these variables and AD.

### Comparison of SMART-Pred With Other AI Tools for AD Diagnostics

SMART-Pred offers a unique approach to AD diagnostics by employing handwriting analysis as a noninvasive biomarker, distinguishing itself from other AI tools in the field. Unlike most existing tools that use cognitive tests, magnetic resonance imaging scans, or blood-based markers, SMART-Pred leverages the DARWIN dataset, which includes handwriting samples from 174 participants (89 patients with AD and 85 healthy controls) across 25 tasks, identifying critical predictors like *air_time* and *paper_time*. This focus provides a novel, cost-effective method for early AD detection. The tool integrates 10 diverse ML models, including random forest, AdaBoost, and neural network, within a user-friendly R Shiny application, democratizing access to advanced AI for nonspecialists and enabling robust performance comparison and customization. With neural network achieving 91% accuracy and SVM achieving 89% accuracy on the test set of the reduced feature model, SMART-Pred surpasses many clinical diagnostic tools (typically having around 81% accuracy) using noninvasive data, and its emphasis on interpretability through variable importance ranking (eg, air_time and paper_time) via the “varImp()” function in R aids physician acceptance and hypothesis formulation.

SMART-Pred stands out due to its reliance on accessible handwriting data compared to resource-intensive methods like magnetic resonance imaging scans or genetic testing used by tools, such as the USC model (approximately 85% accuracy) [180] and the Cambridge AI tool (82% accuracy for progression prediction) [45]. While a quantum ML approach has reported an exceptional 99.89% accuracy, its practical application is limited by computational complexity [181]. On the other hand, SMART-Pred prioritizes ease of use and scalability. Unlike the UCSF model, which predicts AD risk up to 7 years in advance using clinical records (72% accuracy), SMART-Pred focuses on immediate diagnostic classification for direct clinical application. Its ability to bridge complex AI algorithms with user-friendly interfaces addresses a critical gap in clinical adoption, and this is further enhanced by its focus on interpretability, a challenge for many AI models. By pinpointing specific handwriting features as predictors, SMART-Pred not only facilitates early detection but also contributes to understanding potential risk factors, paving the way for tailored interventions.

The interplay between AI interpretability and physician acceptance is a key factor in integrating AI systems into health care. SMART-Pred, a multifaceted R Shiny AI-based predictive modeling application, demonstrated robust predictive power in AD diagnosis using the DARWIN dataset. Incorporating 10 ML algorithms with tunable parameters, it achieved optimal results, with ensemble models like random forest and AdaBoost excelling across evaluation metrics. This provides an efficient, customizable approach to AI modeling, particularly for complex datasets and disease diagnosis, which is extendable to other classification-based scenarios. Its ability to identify potential risk factors makes it valuable for guiding hypothesis formulation and experimentation across health care domains. Research indicates that physicians are more likely to adopt AI technologies when the reasoning behind insights is clear, especially in complex fields like AD diagnosis and management [182-184].

Future studies could focus on clinical assessments to evaluate whether top variables like *air_time* and *paper_time* are sufficient for AD diagnosis, strengthening the synergy between AI outcomes and experimental findings. Additionally, SMART-Pred could be integrated into electronic health record systems to identify biomarkers of successful aging, enhance disease diagnosis and surveillance, and analyze medical records, addressing public health concerns through data-driven insights and personalized medicine strategies. The use of smart home data for predicting AD symptoms has also shown promise, highlighting the potential of diverse data sources in AD research, with SMART-Pred’s handwriting analysis offering a complementary approach.

SMART-Pred demonstrated competitive performance compared to recent AI models for AD prediction. Its unique use of handwriting analysis and accessibility as an R Shiny application contribute significantly to the field. Future work could also explore integrating multiple data modalities to enhance its predictive accuracy and robustness. Additional clinical research is needed to evaluate the practical applicability of the top-ranked variables (*air_time* and *paper_time*) in real-world AD risk assessment and diagnosis.

A comprehensive and comparative analysis of the performance of AI tools for AD detection and prediction is provided in Table 6.

**Table 6.** Comparative performance of AI^a^ tools for AD^b^ detection and prediction.

AI tool	Primary data source	Accuracy	Key features/differentiators
SMART-Pred^c^ (our study)	Handwriting analysis (DARWIN^d^ dataset)	91.00% (neural network) with 95% CI: 0.79‐0.97), AUC=94%	Multi-algorithm R Shiny application (10 ML models) with automated feature selection, explainable AI (SHAP), and cross-validation; noninvasive, cost-effective, user-friendly, generalizable framework applicable to diverse biomarkers and deployable beyond handwriting analysis
Cambridge AI tool [45]	Cognitive tests, MRI^e^ scans	82.00% (progression prediction)	Predicts AD progression within 3 years; 3 times more accurate than standard clinical markers
USC model [180]	MRI scans, genetic data, biomarkers	85.00% (CNN^f^) with control and MCI^g^/AD groups	Uses deep learning for high accuracy in brain scan analysis; integrates diverse data types
Quantum ML^h^ approach [181]	Unspecified (advanced computational methods)	99.89%	Exceptional accuracy but limited by complexity and resource needs; less practical for immediate use
Deep learning CNN [185]	Handwriting analysis (DARWIN dataset)	90.40%	Proposes a novel CNN as a cheap, fast, and accurate solution with a high accuracy, but with no XAI^i^
Particle swarm optimization (PSO) algorithm [186]	Handwriting analysis (DARWIN dataset)	90.57% (LR^j^, AdaBoost^k^, and RF^l^, with RF dominating the accuracy battle with the best feature dimension of 20)	Uses a hybrid ML model for predicting AD using PSO-based feature selection, but with no XAI.
ML [73]	Handwriting analysis (DARWIN dataset)	85.29% (RF)	Proposes a single classifier merging the features from the best tasks, but with no XAI.
ML [187]	DARWIN dataset analysis	82.43% (RF)	Uses several ML techniques with selected tasks of different types and complexities to evaluate the healthy status of patients and a combination of different tasks, but with no XAI
ML [188]	Handwriting analysis (DARWIN dataset)	85% (XGBoost^m^)	Uses several ensemble techniques by implementing a 5-fold cross-validation that ensured robust and unbiased results, but with no XAI
ML [189]	Handwriting analysis (DARWIN dataset)	85.86% (LR)	Uses several ML techniques involving extensive hyperparameter tuning, but with no XAI

a AI: artificial intelligence.

b AD: Alzheimer disease.

c SMART-Pred: Shiny Multi-Algorithm R Tool for Predictive Modeling.

d DARWIN: Diagnosis Alzheimer WIth Handwriting.

e MRI: magnetic resonance imaging.

f CNN: convolutional neural network.

g MCI: mild cognitive impairment.

h ML: machine learning.

i XAI: explainable artificial intelligence.

j LR: linear regression.

k AdaBoost: adaptive boosting.

l RF: random forest.

m XGBoost: extreme gradient boosting.

### Practical Design Implications of SMART-Pred

The implementation of SMART-Pred, an AI-based tool for early AD detection via handwriting analysis, holds transformative practical design implications across clinical diagnostics, health care technology, and neurodegenerative research. In clinical settings, SMART-Pred provides a noninvasive, cost-effective screening option, achieving up to 91% accuracy with the neural network model (with 95% CI: 0.79‐0.97 and AUC=94%), which can be seamlessly integrated into primary care where access to advanced neuroimaging or cerebrospinal fluid testing is limited. Using simple handwriting tasks from the DARWIN dataset, the application enables early identification of motor-cognitive decline, potentially reducing diagnostic delays, especially in underserved populations. This supports value-based care by enhancing efficiency and patient outcomes, aligning with broader trends of AI adoption in health care, and prioritizing patients for further evaluation or early interventions.

Beyond clinical use, SMART-Pred influences health care technology by showcasing a user-friendly R Shiny interface that automates complex ML workflows (data preprocessing, model training, and evaluation) for nonspecialist clinical researchers and providers while addressing challenges like infrastructure compatibility and algorithmic bias. Its focus on explainability through feature importance ranking (*air_time* and *paper_time*) promotes transparency, building trust among clinicians who are wary of black-box systems. In neurodegenerative research, SMART-Pred inspires exploration of noninvasive biomarkers for other disorders like Parkinson disease and Huntington disease, and future validation on datasets like ADNI could lead to hybrid models combining handwriting with imaging or genetic data for enhanced precision. By prioritizing clinical utility, SMART-Pred supports future AI development in radiology and beyond, encouraging interdisciplinary collaboration and clinician involvement in design to address real-world health care needs and drive innovation in diagnostics.

### Limitations and Future Work

The SMART-Pred tool has demonstrated promising capabilities for AD prediction through handwriting analysis, but it faces a few critical limitations in its basic version. Computationally, its exclusive reliance on CPU processing within the R environment restricts parallel computing capabilities, leading to inefficiencies when handling large datasets or complex ML algorithms like neural network [190]. This architectural constraint directly impacts scalability, particularly for resource-intensive tasks such as hyperparameter optimization and multimodal data integration. A fundamental reproducibility challenge stems from the inability to save key outputs (model metrics, ROC curves, and trained model states), hindering longitudinal studies and comparative analyses [191-194]. These limitations collectively restrict the tool’s utility in real-world clinical workflows.

To address these issues, the authors propose an advanced version integrating Python-based frameworks like TensorFlow for GPU-accelerated neural network models and enhanced computational efficiency. Planned upgrades include flexible hyperparameter optimization, automated parameter distribution analysis, and comprehensive result-saving functionalities. Future validation will expand beyond the DARWIN dataset to incorporate multimodal data from the ADNI, encompassing neuroimaging, genetic, and biochemical biomarkers. This broader validation aims to assess generalizability across diverse populations and clinical settings while enabling cross-modal biomarker comparisons, a critical step toward establishing SMART-Pred as a clinically actionable tool for early AD detection.

### Comparison With Prior Work

Our study on SMART-Pred aligns with the growing trend of using AI for the early detection and prediction of AD. The performance of our model (91% accuracy with neural network, 95% CI: 0.79‐0.97; AUC=94%) compares favorably with recent advancements in the field. Table 7 presents a concise comparison of studies that used ML, explainable AI, and the DARWIN dataset for AD detection.

**Table 7.** Comparative analysis of ML^a^ and XAI^b^ studies using the DARWIN^c^ dataset for Alzheimer disease detection.

Dataset	ML techniques used	XAI methods applied	Key insights and features identified	Performance metrics
DARWIN dataset analysis [195]	LR^d^, RF^e^, SVM^f^, and EBM^g^	SHAP^h^ and EBM interpretability	“air_time” (in-air movement duration) identified as the most decisive feature group	89% (SD 5%) accuracy with EBM on the reduced dimensionality dataset
DARWIN dataset analysis [196]	ML and stacking	SHAP XAI features importance and direction of influence	“air_time” and “paper_time” (pen contact duration) features dominate the feature importance analysis using SHAP	88.57% accuracy with the stacking ensemble model
DARWIN dataset analysis [197]	LR, AdaBoost^i^, XGBoost^j^, KNN^k^, RF, SVM, etc	LIME^l^ and ELI5^m^	“air_time” and “paper_time” features were the most important features using ELI5	91.00% accuracy with RF
SMART-Pred^n^ (current study)	LR, AdaBoost, XGBoost, KNN, RF, SVM, neural network, etc	SHAP features importance and direction of influence	“air_time” and “paper_time” as primary biomarkers using SHAP XAI.	91.00% accuracy with neural network; 95% CI 0.79‐0.97; AUC=94%

a ML: machine learning.

b XAI: explainable artificial intelligence.

c DARWIN: Diagnosis Alzheimer WIth Handwriting.

d LR: logistic regression.

e RF: random forest.

f SVM: support vector machine.

g EBM: explainable boosting machine.

h SHAP: Shapley Additive Explanations.

i AdaBoost: adaptive boosting.

j XGBoost: extreme gradient boosting.

k KNN: k-nearest neighbors.

l LIME: Local Interpretable Model-Agnostic Explanations.

m ELI5: Explain Like I’m 5.

n SMART-Pred: Shiny Multi-Algorithm R Tool for Predictive Modeling.

The comparative analysis revealed 3 critical insights. First, the studies demonstrate feature consensus, identifying temporal handwriting features like “air_time” as pivotal predictors, thereby validating their biological relevance to AD motor-cognitive decline through their association with prefrontal cortex dysfunction and motor planning deficits. Second, SMART-Pred represents a methodological evolution from prior work by expanding model diversity (10 algorithms vs up to a maximum of 7 algorithms in earlier studies), incorporating neural networks to capture complex nonlinear patterns in handwriting dynamics, and advancing biomarker identification through dual focus on “air_time” (in-air movement duration) and “paper_time” (pen contact duration) rather than single-feature analysis. Third, despite architectural differences, SMART-Pred classifiers performed very well (with neural network achieving an accuracy of 91% [95% CI 0.79-0.97]), highlighting the DARWIN dataset’s robustness for handwriting-based diagnosis while indicating that model selection impacts biomarker discovery more than absolute accuracy in this domain. This progression from single-feature interpretation to multi-algorithm, multibiomarker analysis reflects an intentional shift toward clinical translatability, balancing explainability with predictive power.

### Conclusion of the Study

The SMART-Pred R Shiny application represents a significant advancement in AI-based predictive modeling for medical diagnostics, offering a user-friendly interface and comprehensive algorithm selection. This tool holds value for researchers and clinicians in personalized medicine and public health surveillance, demonstrating potential to enhance early detection and screening strategies for conditions such as AD and Parkinson disease. By bridging the gap between computational modeling and clinical application, SMART-Pred exemplifies the growing trend of leveraging interactive data visualization and AI-powered analysis in health care settings.

Future development of SMART-Pred will focus on clinical validation and address the challenges associated with imbalanced datasets. The roadmap includes expanding algorithmic support to encompass advanced ML techniques, image recognition architectures, and analysis capabilities for diverse data types such as text, time series, and graph data. Additionally, plans to improve visualization capabilities and user interface design aim to increase accessibility and power. These ongoing enhancements are designed to create a more flexible and comprehensive platform for data analysis across various scientific domains, solidifying SMART-Pred’s role in advancing predictive modeling in health care and beyond.

## Supplementary material

10.2196/70272 Multimedia Appendix 1 SMART-Pred (Shiny Multi-Algorithm R Tool for Predictive Modeling) application and data.

10.2196/70272 Multimedia Appendix 2 Shapley Additive Explanations analysis results for the additional machine learning classifiers.

## Abbreviations

AD: Alzheimer disease
AdaBoost: adaptive boosting
ADNI: Alzheimer’s Disease Neuroimaging Initiative
ADRD: Alzheimer disease and related dementias
AI: artificial intelligence
AUC: area under the curve
DARWIN: Diagnosis Alzheimer With Handwriting
KNN: k-nearest neighbors
PCA: principal component analysis
ROC: receiver operating characteristic
SHAP: Shapley Additive Explanations
SMART-Pred: Shiny Multi-Algorithm R Tool for Predictive Modeling
SVM: support vector machine
XGBoost: extreme gradient boosting
