# Supplementary material for: Artificial Intelligence–Enhanced Multi-Algorithm R Shiny Application for Predictive Modeling and Analytics: Case Study of Alzheimer Disease Diagnostics
Source: JMIR Aging. 2025 Dec 30;8:e70272. doi: 10.2196/70272 (PMC12752916; doi:10.2196/70272)
Supplement: Multimedia Appendix 2 [file aging-v8-e70272-s002.docx]

**APPENDIX**


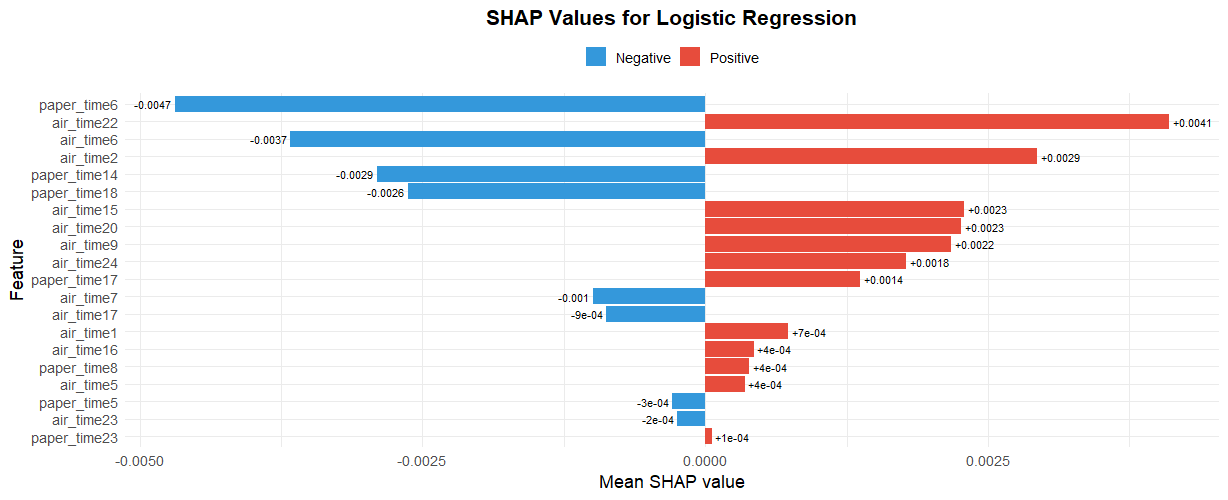


1. **SHAP Analysis using Logistic Regression Classifier**


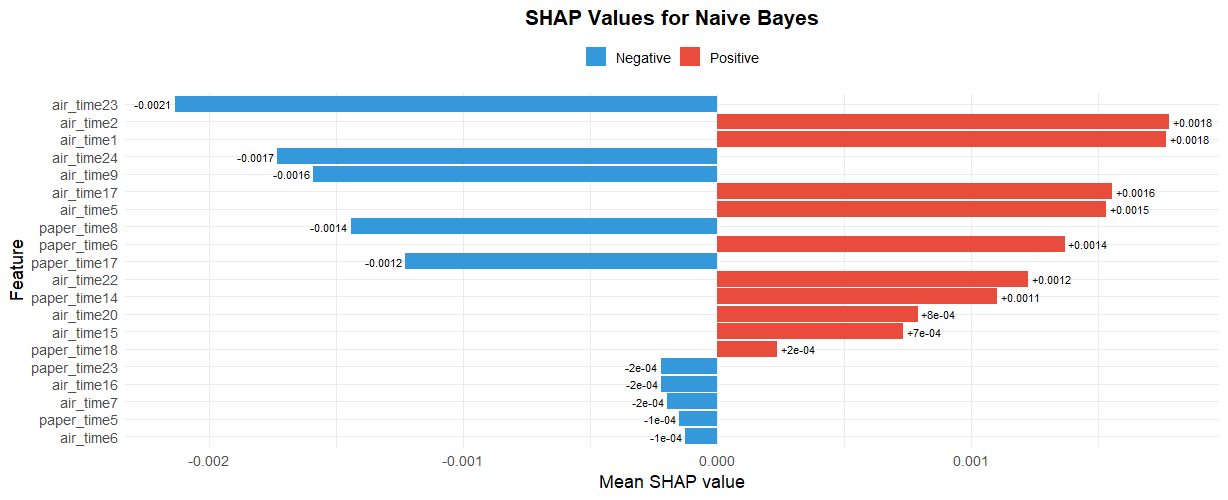


1. **SHAP Analysis using Naïve Bayes Classifier**


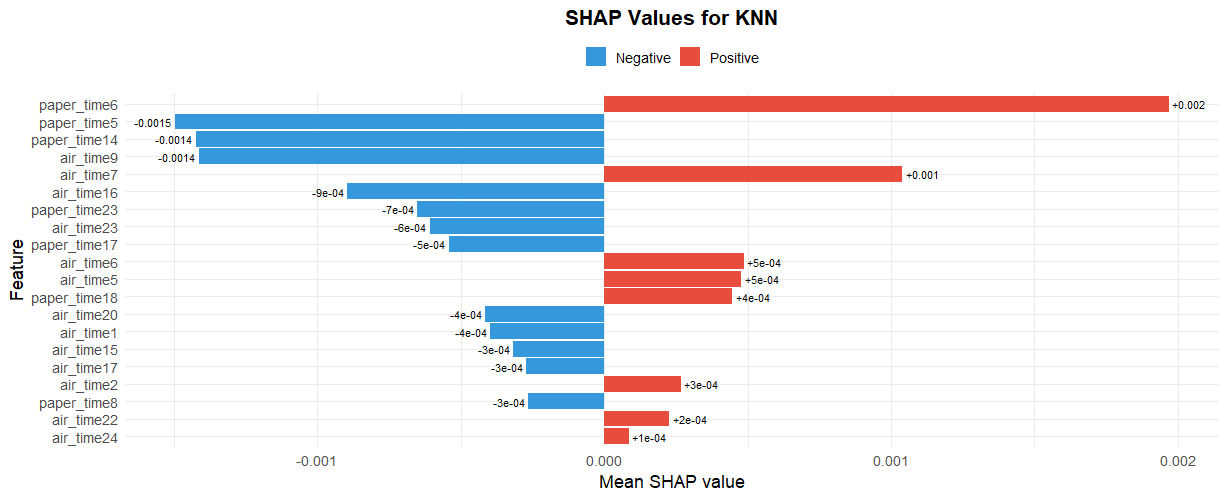


1. **SHAP Analysis using KNN Classifier**


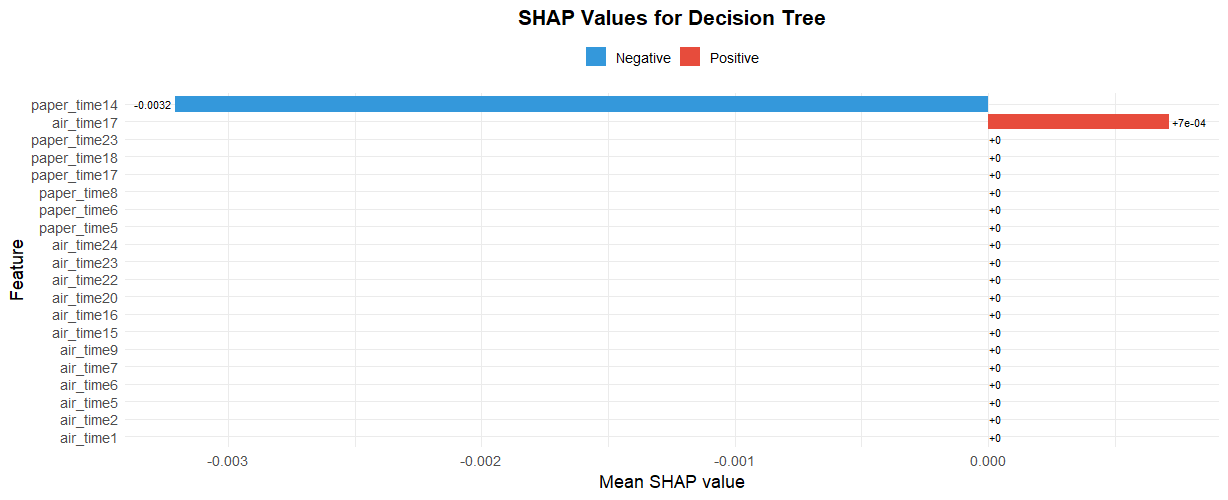


1. **SHAP Analysis using Decision Tree Classifier**


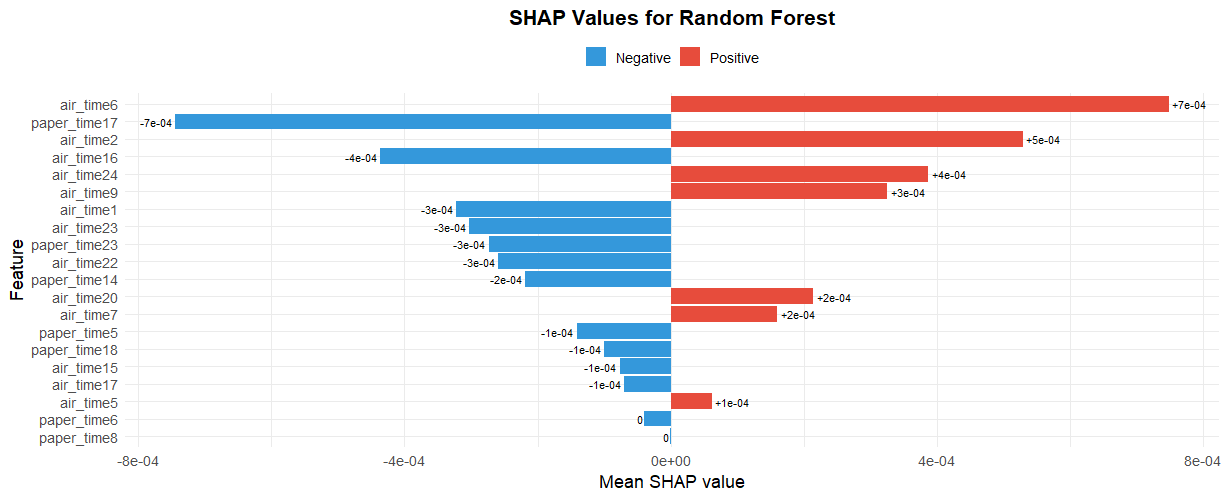


1. **SHAP Analysis using Random Forest Classifier**


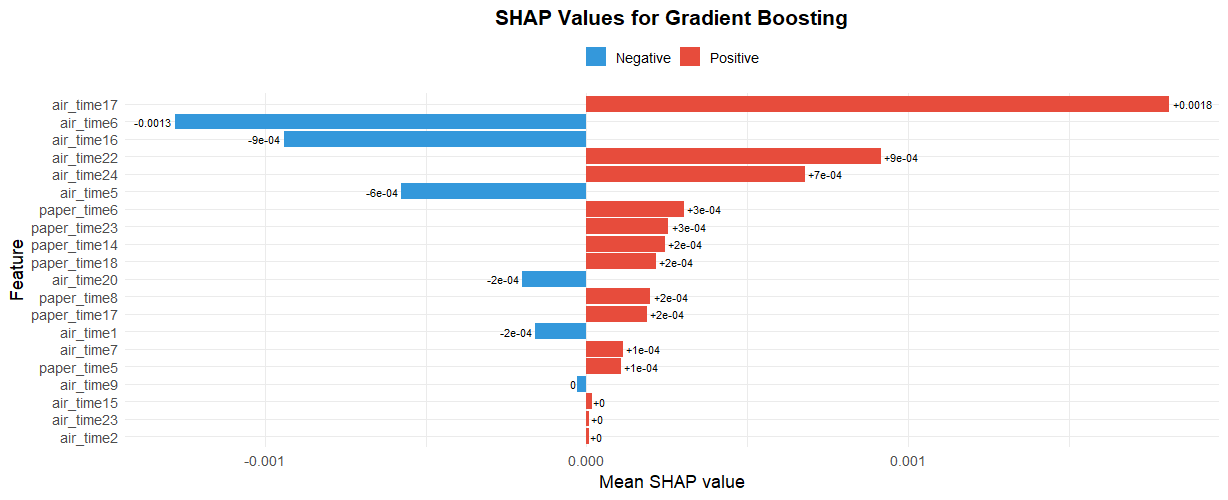


1. **SHAP Analysis using Gradient Boosting Classifier**


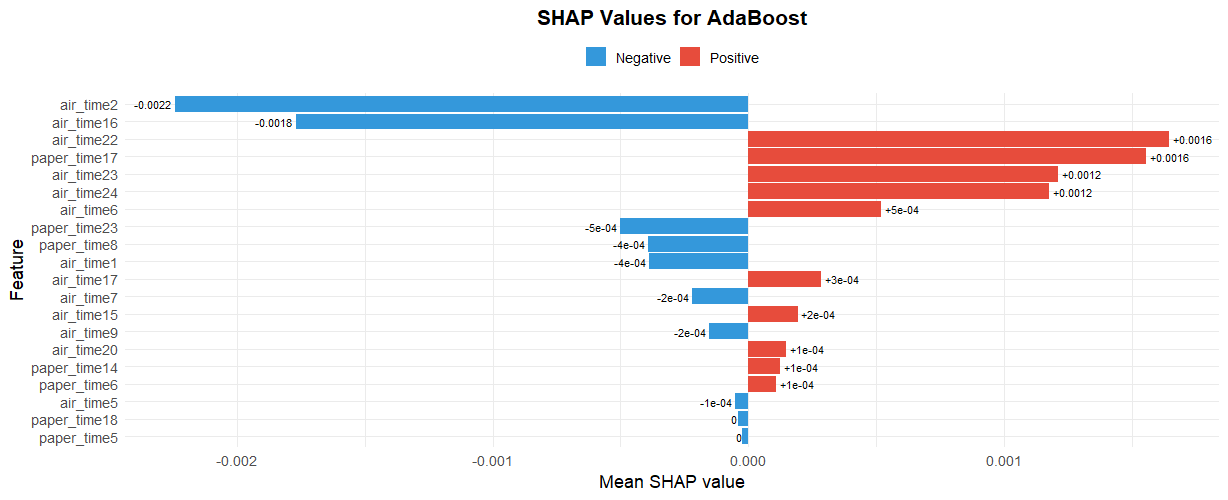


1. **SHAP Analysis using AdaBoost Classifier**


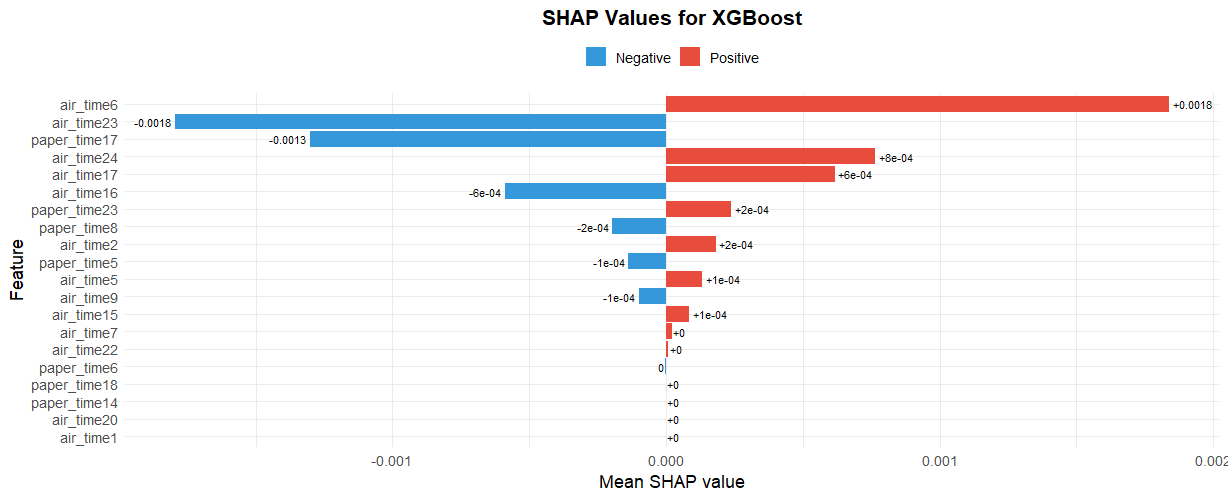


1. **SHAP Analysis using XGBoost Classifier**

**Multimedia Appendix 2. Shapley Additive Explanations analysis results for the additional machine learning classifiers.** This figure presents the SHAP (SHapley Additive exPlanations) summary plots for the eight additional machine learning classifiers evaluated in the study. Each subplot illustrates the contribution of individual features to the predictive output of a specific model, based on their SHAP values. The horizontal axis in each plot represents the SHAP value, indicating the magnitude and direction of each feature’s impact on the model’s predictions. Features are ordered by their overall importance, with those exerting the strongest influence appearing at the top. Color gradients denote the relative value of each feature (from low to high), facilitating interpretation of whether increased or decreased feature values are associated with positive or negative model predictions. Together, these results provide a comprehensive, model-agnostic assessment of feature importance across diverse classifiers, highlighting consistent and divergent predictors of the outcome variable based on the SHAP interpretability framework employed in the manuscript.
